# Development and Evaluation of a Fluorescent Antibody-Drug Conjugate for Molecular Imaging and Targeted Therapy of Pancreatic Cancer

**DOI:** 10.1371/journal.pone.0157762

**Published:** 2016-06-23

**Authors:** Steve Knutson, Erum Raja, Ryan Bomgarden, Marie Nlend, Aoshuang Chen, Ramaswamy Kalyanasundaram, Surbhi Desai

**Affiliations:** 1 Department of Research and Development, Thermo Fisher Scientific, Rockford, Illinois, United States of America; 2 Department of Biomedical Sciences, University of Illinois College of Medicine, Rockford, Illinois, United States of America; Monash University, AUSTRALIA

## Abstract

Antibodies are widely available and cost-effective research tools in life science, and antibody conjugates are now extensively used for targeted therapy, immunohistochemical staining, or *in vivo* diagnostic imaging of cancer. Significant advances in site-specific antibody labeling technologies have enabled the production of highly characterized and homogenous conjugates for biomedical purposes, and some recent studies have utilized site-specific labeling to synthesize bifunctional antibody conjugates with both imaging and drug delivery properties. While these advances are important for the clinical safety and efficacy of such biologics, these techniques can also be difficult, expensive, and time-consuming. Furthermore, antibody-drug conjugates (ADCs) used for tumor treatment generally remain distinct from conjugates used for diagnosis. Thus, there exists a need to develop simple dual-labeling methods for efficient therapeutic and diagnostic evaluation of antibody conjugates in pre-clinical model systems. Here, we present a rapid and simple method utilizing commercially available reagents for synthesizing a dual-labeled fluorescent ADC. Further, we demonstrate the fluorescent ADC’s utility for simultaneous targeted therapy and molecular imaging of cancer both *in vitro* and *in vivo*. Employing non-site-specific, amine-reactive chemistry, our novel biopharmaceutical theranostic is a monoclonal antibody specific for a carcinoembryonic antigen (CEA) biomarker conjugated to both paclitaxel and a near-infrared (NIR), polyethylene glycol modified (PEGylated) fluorophore (DyLight™ 680-4xPEG). Using *in vitro* systems, we demonstrate that this fluorescent ADC selectively binds a CEA-positive pancreatic cancer cell line (BxPC-3) in immunofluorescent staining and flow cytometry, exhibits efficient internalization kinetics, and is cytotoxic. Model studies using a xenograft of BxPC-3 cells in athymic mice also show the fluorescent ADC’s efficacy in detecting tumors *in vivo* and inhibiting tumor growth more effectively than equimolar amounts of unconjugated drug. Overall, our results demonstrate that non-selective, amine-targeting chemistry is an effective dual-labeling method for synthesizing and evaluating a bifunctional fluorescent antibody-drug conjugate, allowing concurrent detection, monitoring and treatment of cancer.

## Introduction

Antibodies are highly effective tools in contemporary human medicine, particularly in the diagnosis and treatment of cancer. Antibodies can be intrinsically therapeutic, or conjugated to different molecules for a variety of biomedical applications [1]. For decades, radioactive, fluorescent, and enzyme labels covalently attached to antibodies, antibody fragments, or other affinity biomolecules have been routinely used in non-clinical research for antigen detection [2]. More recent research has also highlighted the enormous potential of antibody conjugates as pharmaceutical tools for diagnosing disease. Known as molecular, optical, or simply “*in vivo*” imaging, this modality involves a probe circulating in the body until it binds a specific target for visualization using specialized instrumentation [3, 4]. Antibodies can also be coupled to therapeutic small molecules for drug delivery applications. These antibody-drug conjugates (ADCs) target individual tissues or cells through disease biomarker recognition, localizing the drug to these areas. Upon cellular internalization, the ADC releases its drug payload to kill the cell [5–8]. Targeted therapy utilizing ADCs is highly promising, with two ADCs, brentuximab vedotin (Adcetris) and trastuzumab emtansine (Kadcyla), currently approved by the FDA, with at least 30 more in various phases of development and clinical trials [9,10]. The current commercial availability of monoclonal antibodies targeting innumerable antigens of interest has also grown exponentially, presenting an unprecedented opportunity to study the therapeutic and diagnostic potential of a wide range of antibodies [11].

Whether labeled with imaging agents for molecular diagnostics or conjugated to drugs for targeted therapy, chemical modification strategies are necessary to couple these components to a protein or antibody base. There are myriad protein bioconjugation techniques currently available, employing a wide variety of strategies to label amino acids, carbohydrates, or other moieties present on a protein of interest. Of these classical methods, two of the most effective and well-characterized are modification of cysteine and lysine residues [12]. In native proteins and antibodies, oxidized cysteine pairs (i.e. disulfide bonds) can be easily reduced to generate free thiols for alkylation reactions with iodoacetamide or maleimide reagents [13]. Primary amine groups present on surface-accessible lysines (as well as the N-terminus) also react prodigiously with a variety of compounds including carbodiimides, aldehydes, imidoesters, and most commonly, N-Hydroxysuccinimide Esters (NHS Esters), forming stable amide bonds [14]. Indeed, heterobifunctional crosslinkers exploiting both cysteine and lysine modification chemistries are almost exclusively used to form antibody-drug linkages in ADCs [15–17]. For example, brentuximab vedotin (Adcetris) is comprised of an anti-CD30 monoclonal antibody conjugated to the antimitotic compound monomethyl auristatin E (MMAE), using a maleimide-based heterobifunctional linker [18, 19]. Similarly, several ADCs in clinical development also utilize cysteine-directed maleimide chemistry to conjugate various therapeutic antibodies to their respective cytotoxic payloads, including glembatumumab vedotin, pinatuzumab vedotin, and PSMA-ADC [20–22]. Conversely, trastuzumab entamsine (Kadcyla) relies on an NHS ester-based bifunctional crosslinker, succinimidyl *trans*-4-(maleimidylmethyl)cyclohexane-1-carboxylate (SMCC), to covalently attach the cytotoxic small molecule DM1, a maytansinoid antimitotic microtubule disruptor, to the therapeutic antibody Herceptin through exposed surface lysines [23]. Analagously, various ADCs in the clinical pipeline also use this amine-reactive lysine chemistry for drug loading, including milatuzumab-doxorubicin, inotuzumab ozogamicin, indatuximab ravtansine, coltuximab ravtansine, and lorvotuzumab mertansine [24–28].

While cysteine and lysine targeting conjugation methods are simple and demonstrably effective in these aforementioned examples, these techniques are also largely considered “regioselective”, “nonselective”, or “non-site-specific”, resulting in a heterogenous distribution of differentially labeled protein conjugates. For instance, trastuzumab emtansine is molecularly comprised of a mixture of ADCs with drug-to-antibody ratios (DARs) ranging from 0–8, with an overall average DAR of 3.5 [29]. This distribution is potentially problematic in a pharmacological context, where ADCs with particularly high DARs exhibit instability, insolubility, poor pharmacokinetics, and increased toxicity [30–32]. New advancements in bioconjugation research have attenuated several of these issues by enabling “site-specific” labeling at precise locations on proteins. These various methods include the insertion of specialized amino acid conjugation sites (e.g. engineered cysteines or unnatural amino acids), the use of enzymes to catalyze antibody-drug linkage at specific residues, or the utilization of next generation maleimide reagents for simultaneous disulfide re-bridging and site-specific labeling of antibody conjugates [33–45]. In addition to yielding more homogenous distributions of conjugates, these techniques have also been shown to result in ADCs with enhanced pharmacological properties, improving both the safety and efficacy of these biopharmaceuticals [46]. However, these techniques require multiple, difficult, and time-consuming processing steps, including complete engineering of a recombinant antibody sequence, optimization of an appropriate bioorthogonal cell line expression system, and/or precise control over sequential reduction and re-bridging for antibody labeling. The vast array of promising drugs, antibodies, and cancer targets/biomarkers currently being researched requires simpler methods to dual label antibodies that enable efficient pre-clinical evaluation of molecular diagnostic and therapeutic potential of these combinations.

Paclitaxel is one such promising cytotoxic compound, first purified in the late 1960’s and later characterized in greater detail by Wani, M. C. et al [47]. Similar to other ADC cytotoxic payloads, including DM1 and MMAE, paclitaxel is a mitotic inhibitor that binds and stabilizes microtubules, interrupting normal mitotic spindle assembly and chromosome segregation during G_2_/M phase leading to apoptosis [48]. Despite paclitaxel’s cytotoxic utility in cancer chemotherapy, it is also has poor aqueous solubility, requiring harsh delivery mechanisms to administer it. To attenuate these effects, as well as increasing the potency, efficacy, and solubility of paclitaxel itself, numerous paclitaxel derivatives and prodrugs have been developed, including conjugation to PEG, docosahexaenoic acid, hyaluronic acid, arginylglycylaspartic acid, poly-L-glutamic acid, and albumin [49–55]. Cross-linking paclitaxel to antibodies has also been investigated, with several studies demonstrating the pre-clinical effectiveness of these ADCs for use in cancer treatment [56–59].

Selecting a therapeutic target and/or biomarker is also an important consideration in the design of both ADCs and molecular imaging reagents. Carcinoembryonic antigen (CEA) is a developmental, cell-surface adhesion protein over-expressed in many gastrointestinal, breast, ovarian, lung, and thyroid carcinomas, and is a well established and attractive cancer biomarker [60, 61]. Several reports have shown the effectiveness of targeting CEA in anti-cancer applications [62–65]. There also exists an impressive body of work demonstrating the biomarker’s efficacy as a cancer imaging and diagnostic tool, ranging from radio-labeled CEA antibodies [66–71] to the use of fluorescent labels [72–79]. More recent work has demonstrated the improved optical performance and physiological stability of PEGylated, NIR dyes coupled to CEA-specific antibodies for highly effective molecular imaging of tumors [80, 81].

Presently, the use of antibody conjugates for cancer diagnosis or treatment are distinct molecules with different uses. Thus, there exists a need to unify these applications in the clinical setting. Previous research has demonstrated the indirect fluorescent labeling of ADCs for concurrent imaging of tumors [82]. Other studies have utilized a disulfide re-bridging strategy to achieve dual, site-specific labeling of Herceptin with both a chemotherapeutic drug and a fluorophore, but these studies only assessed efficacy *in vitro* [43–45]. Here, we present an alternative, simple method for synthesizing a dual-labeled fluorescent ADC that is capable of simultaneous targeted therapy and molecular imaging *in vivo*. Given the prior diagnostic and therapeutic promise surrounding these components, we utilized a commercially available CEA mouse monoclonal antibody (Thermo Fisher Scientific #MIC0101, IgG_1_ isotype, clone 1105) for direct conjugation to both paclitaxel and the PEGylated, NIR fluorophore, DyLight™-680-4xPEG. In addition to the synthesis and analytical characterization of this conjugate, we demonstrate the first *in vitro* and *in vivo* evaluation of this type of bifunctional antibody construct for simultaneous treatment and detection of cancer. Further, the method we report here is widely applicable, and can be used to quickly and effectively synthesize a variety of bifunctional antibody and protein conjugates for a multitude of biomedical research applications.

## Results

### Synthesis, Purification, and Characterization

**Fig 1:**
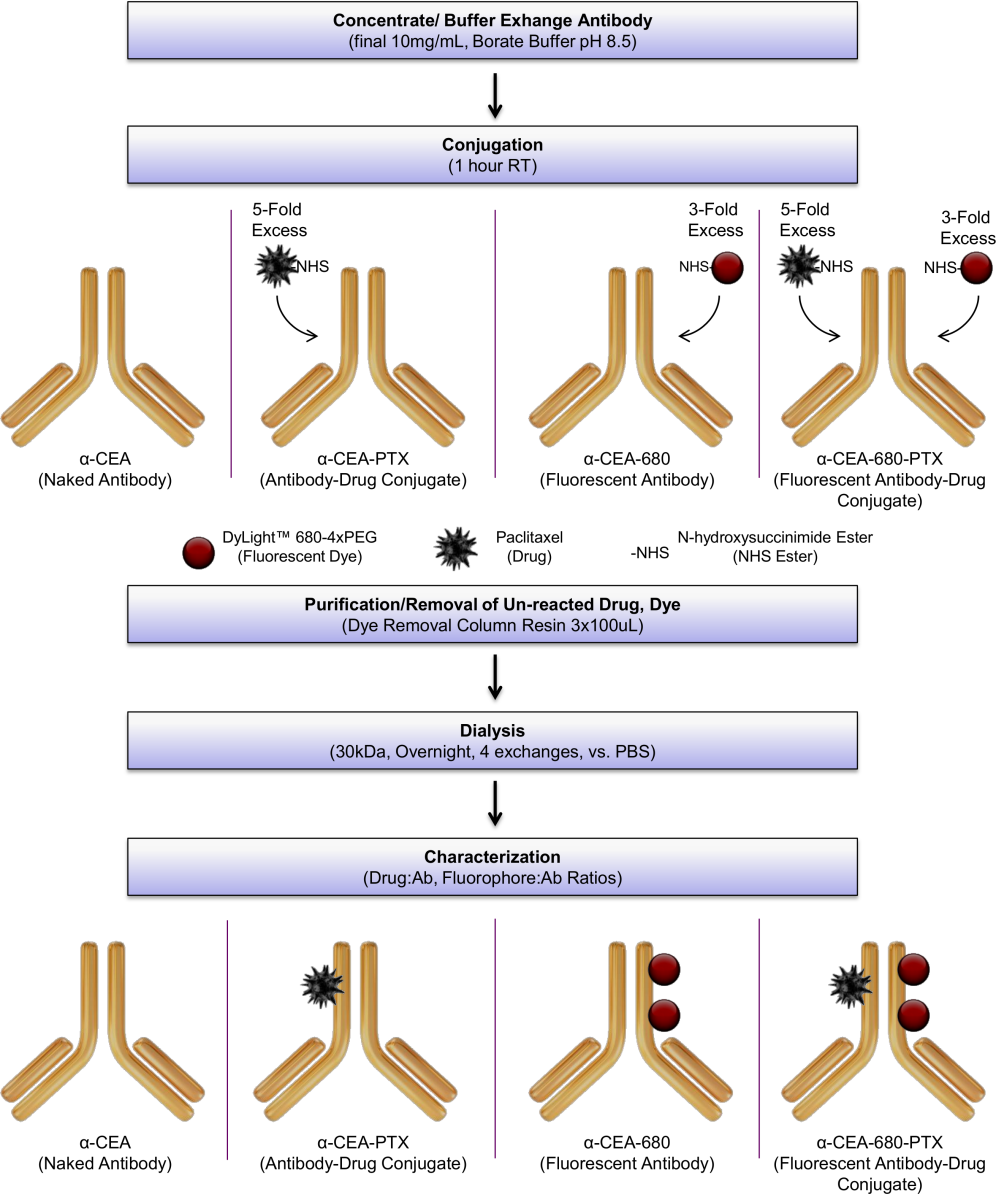
Workflow for Synthesis, Purification, and Characterization of Conjugates. Mouse monoclonal antibody specific for human CEA was used to prepare four samples for subsequent evaluation and comparison. Two mouse IgG_1_ isotype control conjugate samples were also prepared as stated in Table 1. The bottom portion of Fig 1 illustrates approximate characterized loading ratios for both drug and dye in various CEA samples.

Amine-reactive NHS ester chemistry was used to conjugate both paclitaxel and DyLight™-680-4xPEG to an antibody specific for CEA. Paclitaxel was first derivatized into a succinate ester (Paclitaxel-NHS or “PTX-NHS”) using a procedure outlined previously [51]. To effectively evaluate different CEA antibody modifications, four conjugate samples (with and without labels) were synthesized, purified, and characterized as depicted in Fig 1. Two mouse IgG isotype control samples were also prepared to assess specificity of the antibody conjugate/theranostic during *in vitro* fluorescent binding assays. After concentration and buffer exchange, antibodies were reacted with the noted molar excesses of appropriate components. Samples were then purified with the resin included in the Pierce™ Dye Removal Column kit followed by dialysis in PBS. Conjugates were then characterized by UV/Vis analysis for fluorophore:antibody ratio (FAR) estimation. Drug:antibody ratio (DAR) was determined with a modified procedure taken from [58] by hydrolyzing the ester antibody-drug linkage and then quantifying extracted paclitaxel by liquid chromatography. Table 1 summarizes the characterized DARs and FARs for all antibody conjugates used in this study.

**Table 1:** Analytical Characterization of Antibody Samples and Conjugates.

Sample	DAR	FAR
**α-CEA**	NA	NA
**α-CEA-PTX**	1:1	NA
**α-CEA-680**	NA	2.4:1
**α-CEA-680-PTX**	1.2:1	2.1:1
**IgG-680**	NA	2.3:1
**IgG-680-PTX**	1.1:1	2.2:1

Overall, the protocol for conjugating both paclitaxel and a PEGylated fluorophore to the CEA antibody was quite simple using established amine-reactive crosslinking chemistry. For effective comparison, it was desired that the different conjugates had similar DARs and FARs (Table 1). Unexpectedly, we found that the same molar excesses of each component (PTX-NHS or DyLight™ 680-4xPEG-NHS) could be used for every conjugation reaction, whether used alone or mixed together in a single reaction (Fig 1). A 3-fold molar excess of dye resulted in an approximate FAR of 2:1 for each conjugate, while a 5-fold molar excess of drug resulted in a relatively lower DAR of 1:1. These data suggest that the fluorophore labeling chemistry is more efficient, or perhaps more reactive due to differences in accessibility or compound solubility. These results were consistent in multiple CEA antibody and isotype control conjugate preparations in the development of this method, suggesting that the amine-reactive drug and dye either do not compete for the same amino groups, or may preferentially react with distinct sites on the antibody. In addition, these observations are indicative that these procedures can be potentially applied to other antibodies and proteins.

Another unexpected finding during the conjugate purification method development was that the dye removal columns efficiently removed both excess, unreacted dye and drug (verified by high-performance liquid chromatography). While the resin affinity for different fluorophores has been shown, this resin has never been demonstrated to remove other types of small molecules. Since paclitaxel is a large and hydrophobic molecule, this drug may have some properties similar to common fluorophores which are involved in affinity interactions with the dye removal resin. Ultimately, this method enabled us to produce highly pure conjugates in a single purification step.

**Fig 2:**
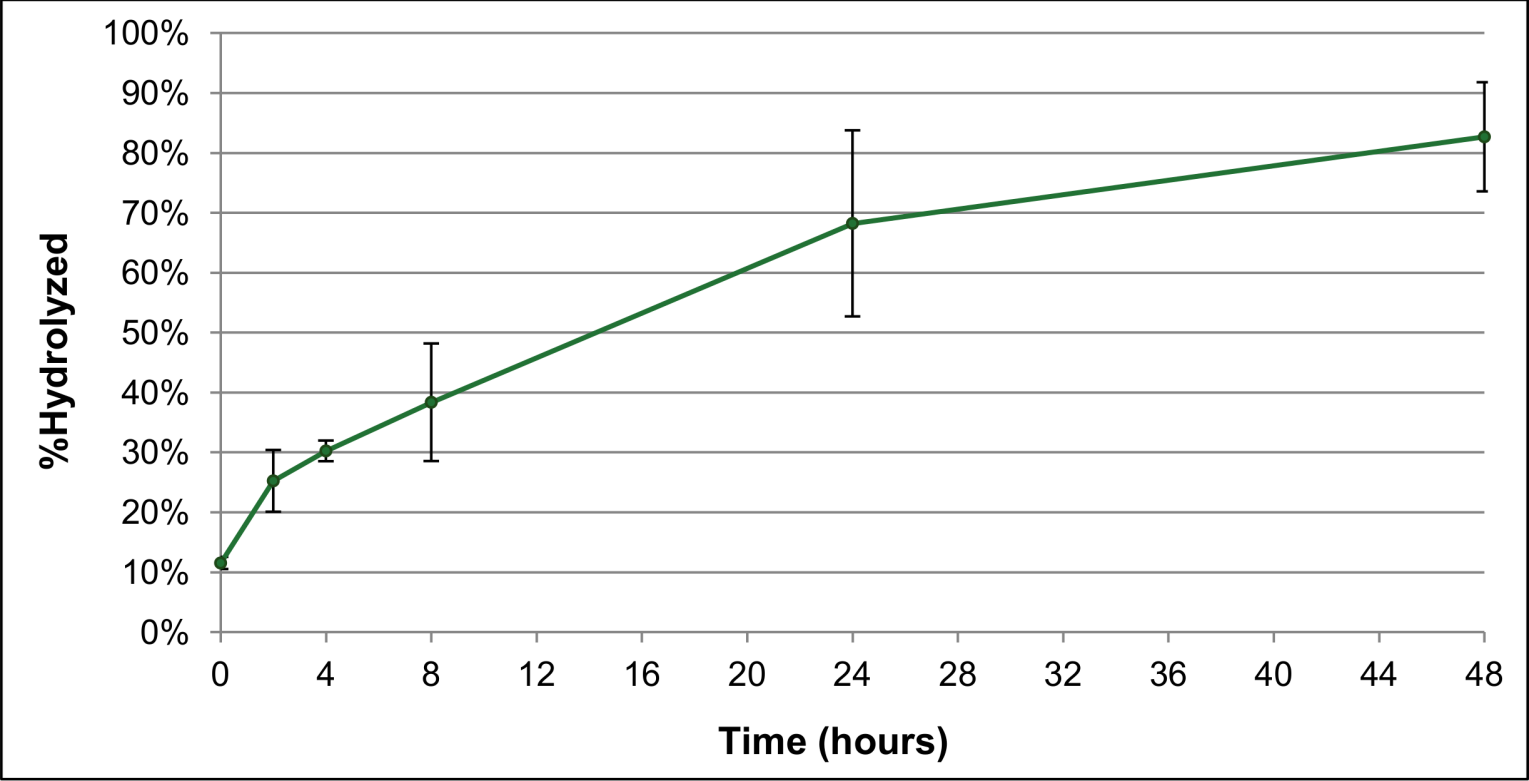
Estimation of Physiological Conjugate Stability *In Vitro*. The fluorescent ADC and hydrolyzed controls were incubated in PBS at 37°C, and liberated paclitaxel was extracted and quantified. Degree of hydrolysis was defined as the ratio of extracted paclitaxel from test samples compared to hydrolyzed controls. Data points represent triplicate extracted samples (n = 3). Error bars denote standard error.

In ADC applications, it is also vital to assess the chemical stability of the antibody-drug linker for eventual pharmacological considerations. Stability of the α-CEA-680-PTX antibody-drug linker (structurally illustrated in [58]) was determined through *in vitro* hydrolysis of the ester bond linkage followed by C18 extraction and analysis of free drug concentration by HPLC. For triplicate samples, the fluorescent ADC was hydrolyzed over time at physiological temperatures and compared to a control sample mixture of the unconjugated antibody and free paclitaxel. Degree of hydrolysis at each time point was defined as the peak area ratio of extracted paclitaxel from test samples compared to hydrolyzed controls. As shown in Fig 2, these data illustrate a time-dependent release of paclitaxel, and the *in vitro* physiological half-life of the conjugate linkage is estimated at 12–16 hours. The stability of the paclitaxel-antibody linkage was also approximated in a previous report, where hydrolytic half-life was determined to be 2–3 hours at physiological conditions [83]. However, this study utilized a radioactive, benzoyl-labeled paclitaxel linker derivative to make these estimates, with distinct chemical and structural differences, so comparisons between these approximations are difficult. In either case, the stability of this particular antibody-drug linker appears to be somewhat labile.

### *In Vitro* Fluorescent Evaluation

**Fig 3:**
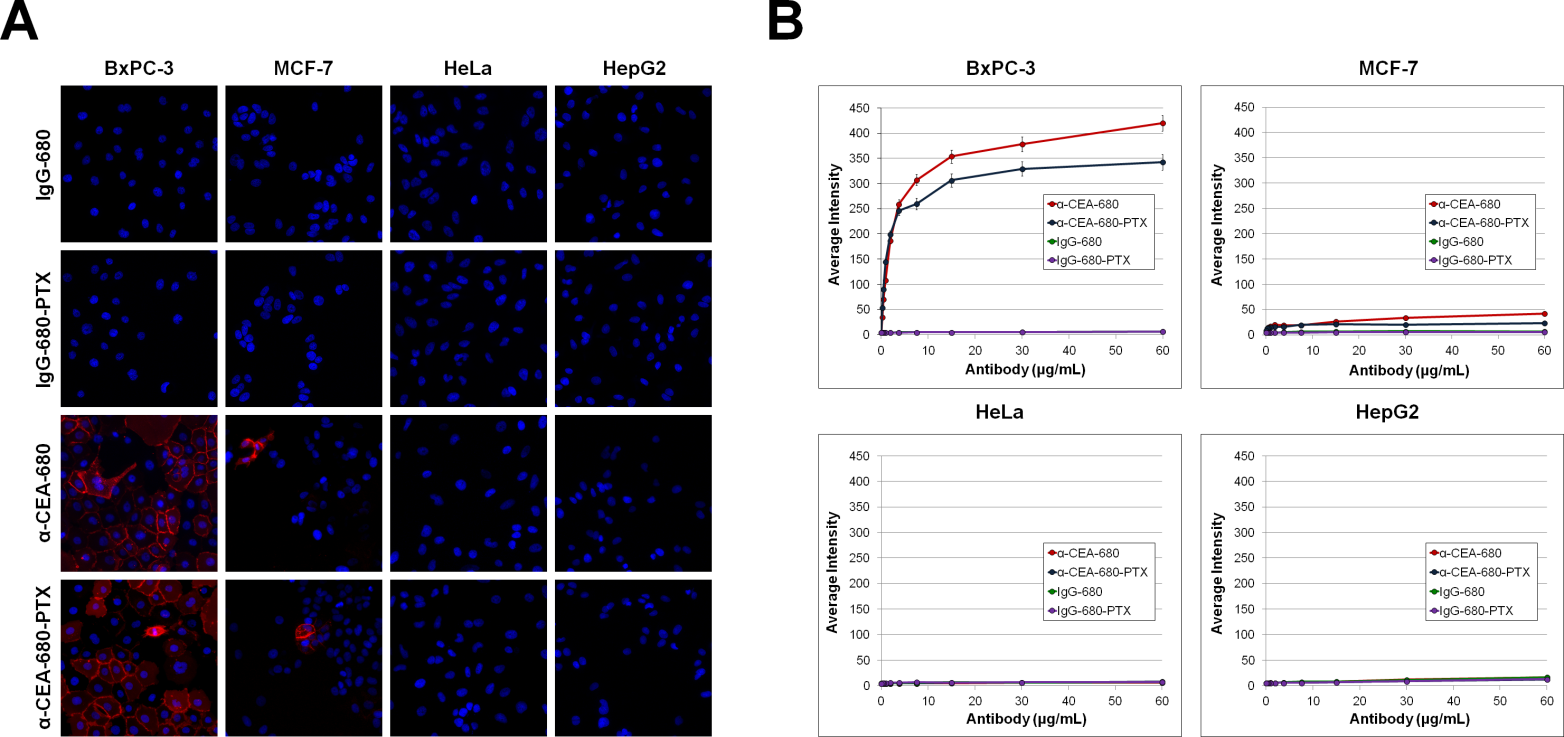
Immunofluorescent Staining of Cell lines with Conjugates and Quantification of Signal. (A) Immunofluorescent images of cell lines stained with various conjugate samples. Two putative CEA-positive cell lines, BxPC-3 and MCF-7, and two control CEA-negative cell lines, HeLa and HepG2, were seeded at 1x10^4^ cells/well in 96-well collagen-coated plates. Cells were immunofluorescently stained with approximately 2ug/mL of α-CEA-680 or α-CEA-680-PTX where noted (red). Nuclei were counterstained with Hoechst dye (blue). Images were obtained on an ArrayScan VTI under 20X magnification and arranged with the HCS View software, Pxlr editor, and Microsoft PowerPoint. (B) Quantification of Immunofluorescent Staining. BxPC-3, MCF-7, HeLa, and HepG2 cell lines were seeded at 1x10^4^ cells/well in 96-well collagen-coated plates. Cell lines were probed with α-CEA-680 (red lines), α-CEA-680-PTX (blue lines), IgG-680 (green lines), or IgG-680-PTX (purple lines) at concentrations of 0 – 60ug/mL. Fluorescent signal intensity was quantified with the ArrayScan VTI and vHCS Scan software. Data points represent average fluorescent intensity (n = 3000 cells), and error bars denote standard error. Error bars are present at every data point, but are very small in some cases.

**Fig 4:**
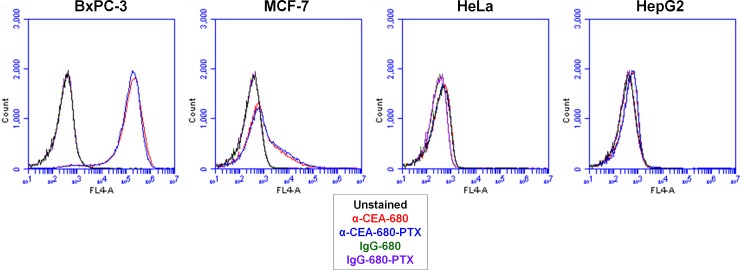
Flow Cytometry Analysis of Cell lines Stained with Conjugates. BxPC-3, MCF-7, HeLa, and HepG2 cells (1x10^6^ in each sample) were probed with either buffer (unstained, black histograms) or a 10ug/mL solution of α-CEA-680 (red histograms), α-CEA-680-PTX (blue histograms), IgG-680 (green histograms), or IgG-680-PTX (purple histograms). Cells were analyzed using the BD Accuri C6 Flow Cytometer and CSampler Workspace software. Each histogram represents 50,000 total events.

One of the major goals of this study was to determine if fluorescent labeling significantly altered ADC function, or if conjugating a drug to a fluorescent antibody would otherwise impede molecular imaging performance. To evaluate this latter idea, various cell lines (two purported CEA-positive cell lines, BxPC-3 and MCF-7, and two CEA-negative control cell lines, HeLa and HepG2) were incubated with several concentrations of α-CEA-680 or α-CEA-680-PTX, and analyzed via immunofluorescence (Fig 3) and flow cytometry (Fig 4) for both specificity and signal performance. In both experiments, all cell lines were also incubated with isotype control conjugates, IgG-680, and IgG-680-PTX, to assess *in vitro* non-specific binding.

In both *in vitro* model systems, fluorescently labeled anti-CEA conjugates showed high reactivity for the CEA-positive BxPC-3 cell line and minimal response to both CEA-negative HeLa and HepG2 cell lines (Figs 3 and 4). Another putative CEA-positive cell line, MCF-7, had only partial staining of a subpopulation of cells in both immunofluorescence and flow cytometry immunoassays in replicate experiments with different sources of the cell line. These results were consistent with previous reports that MCF-7 is heterogeneous for CEA expression [84–86]. In terms of fluorescent performance, α-CEA-680-PTX exhibited slightly lower intensities across a range of concentrations when compared to identical amounts of α-CEA-680 (Fig 4). This difference between the two conjugates is most likely due to the lower FAR of α-CEA-680-PTX compared to α-CEA-680 (Table 1). However, the addition of paclitaxel may also have an effect on the fluorophore fluorescent properties, antibody solubility, or antibody-antigen binding. Regardless of the molecular and/or biochemical reason for this variation, there were no substantial differences observed for *in vitro* fluorescent localization of α-CEA-680-PTX (fluorescent ADC) and α-CEA-680 (fluorescent antibody). As expected, both isotype control conjugates, IgG-680 and IgG-680-PTX, displayed virtually no affinity for any cell lines tested, regardless of concentration or application tested (Figs 3 and 4).

**Fig 5:**
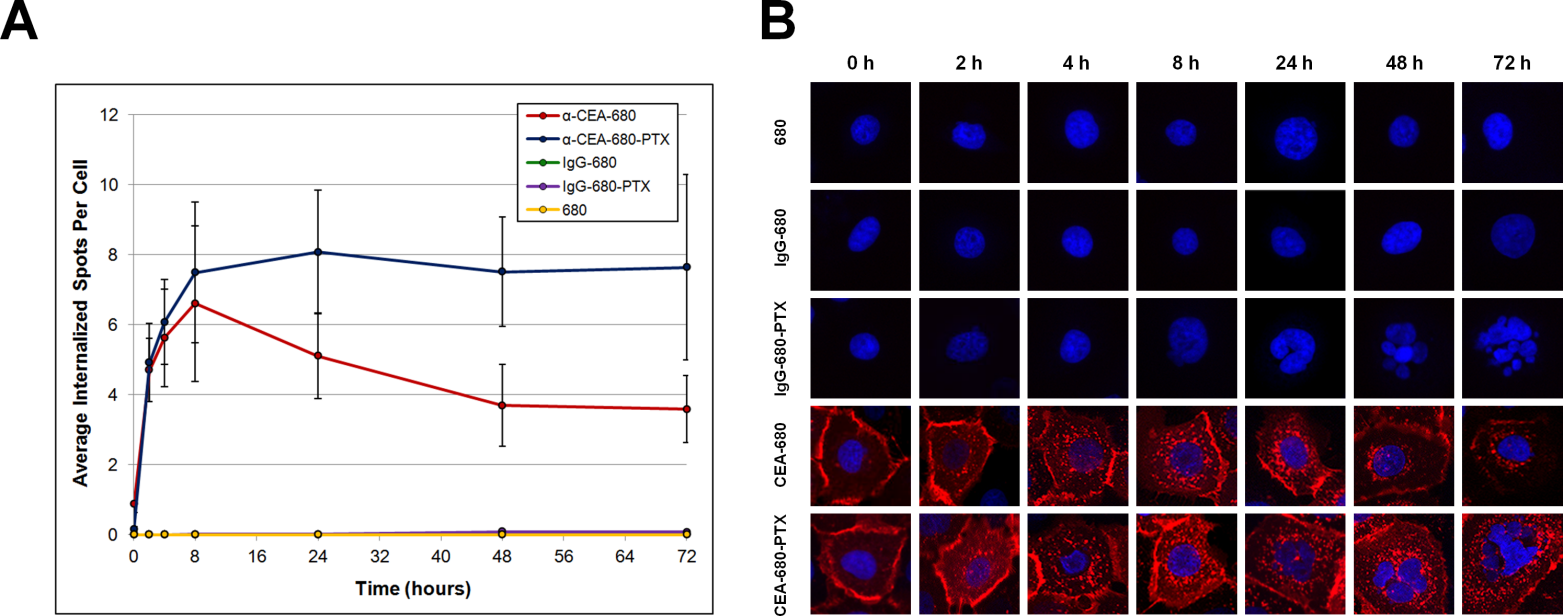
Immunofluorescent Assessment of CEA Internalization. (A) Quantification of internalized CEA “spots”. BxPC-3 cells were seeded at 1x10^4^ cells/well in 96-well collagen-coated plates. Cells were then treated with 46ng/mL free DyLight™ 680-4xPEG or 2ug/mL of IgG-680, IgG-680-PTX, α-CEA-680, or α-CEA-680-PTX diluted in RPMI completed media (all at 46ng/mL effective DyLight™ 680-4xPEG) for the time points indicated. Cells were then washed, fixed, and counterstained with Hoechst dye. Average internalized spots per cell were obtained with the ArrayScan VTI and vHCS Scan software. Data points represent the average number of internalized spots per cell (n = 10,000 cells), and error bars denote standard error. Error bars are present at every data point, but are very small in some cases. (B) Immunofluorescent images depicting representative cells at each time point. Images (20X magnification) were obtained in concert with spot detection analysis on the ArrayScan VTI, and arranged with the vHCS View software, Pxlr editor, and Microsoft PowerPoint.

A further key assessment of ADCs is the estimation of internalization kinetics. After ADCs bind to a specific cell surface receptor antigen, it must be internalized before subsequent lysosomal and/or proteolytic release of the drug intracellularly [87]. Several previous reports have assessed CEA internalization, especially when CEA-positive cells have been probed with antibodies [88–91], CEA-targeting polymeric particles [92, 93] or an anti-CEA immunotoxin conjugate [94]. We have employed a high-throughput, instrument-based immunofluorescent assay to quantitatively detect and measure CEA internalization in BxPC-3 cells probed with various conjugates synthesized in this study. This well-characterized method has been utilized in multiple studies to assess real-time receptor internalization and translocation events in a wide variety of contexts *in vitro* [95–105]. BxPC-3 cells were seeded into 96-well plates, and exposed to either free DyLight™ 680-4xPEG, IgG-680, IgG-680-PTX, α-CEA-680, or α-CEA-680-PTX for incubation times ranging from 0–72 hours. At the time points indicated, cells were washed extensively and then fixed and counterstained. Stained cells were then imaged with the ArrayScan VTI HCS instrument, and the appearance of internalized antibody-bound CEA “spots” over time was quantitatively assessed using the “Spot Detector” algorithm bioapplication in the ArrayScan VTI vHCS Scan software (Fig 5).

It is clear from both panels in Fig 5 that α-CEA-680 and α-CEA-680-PTX underwent progressive internalization from 0–8 hours. CEA internalization was observed as early as 2 hours, with maximal CEA internalization seen approximately 8–24 hours after exposure. Previous studies with other CEA expressing cell lines reported significant, noticeable internalization of antibodies ranging from as early as 5 minutes [88, 90] to 5 hours [89]. Another extensive study evaluating several cell lines, CEA antibody clones, and internalization methods estimated CEA turnover half times at 10–16 hours, with some antibody clones expediting this to as little as 4 hours [91]. Further, while the exact internalization kinetics were unknown in this particular system, a prior report using BxPC-3 cells incubated with CEA-targeting antibody-nanoparticle hybrids observed significant internalization after 30 minutes [93]. Taken together, the internalization kinetics we observed in this study are consistent with these previous assessments.

While we observed internalization for both α-CEA-680 and α-CEA-680-PTX, their kinetic profiles were markedly different (Fig 5A). Although both fluorescent antibodies were steadily internalized from 0–8 hours, the number of detected spots decreased after 8–72 hours for α-CEA-680 but remained constant for α-CEA-680-PTX. One explanation for these data is that viable cells progressively degrade α-CEA-680 and liberate the dye inside vesicles, allowing eventual clearance of the dye through several rounds of endosomal recycling and extracellular release [106]. This is supported by both the observed decrease in overall fluorescent intensity as well as the general translocation of signal to the perinuclear region from 8–72 hours (Fig 5B). It is very unlikely that these results are due to passive diffusion of DyLight™ 680-4xPEG in or out of the cells, as evidenced by the lack of any observed or detected internalization of the free dye by itself.

For α-CEA-680-PTX, the persistent staining of internalized CEA spots is most likely due to the cytotoxic and antimitotic action of paclitaxel itself. Endosomal metabolism has been shown to be dependent on microtubules [107], which may explain why dye release and clearance is slower in cells exposed to PTX. In addition, the onset of apoptosis observed after α-CEA-680-PTX treatment may further inhibit spot degradation. As expected for both isotype control conjugates, IgG-680 and IgG-680-PTX, there was no observed localization or internalization during the entire duration of treatment. However, it is worth noting that treatment with IgG-680-PTX also produced readily apparent, apoptotic nuclear fissure, particularly after prolonged incubation times (24–72 hours), likely explained by the estimated stability characteristics of the antibody-drug linker (Fig 2).

### *In Vitro* Cytotoxic Efficacy

**Fig 6:**
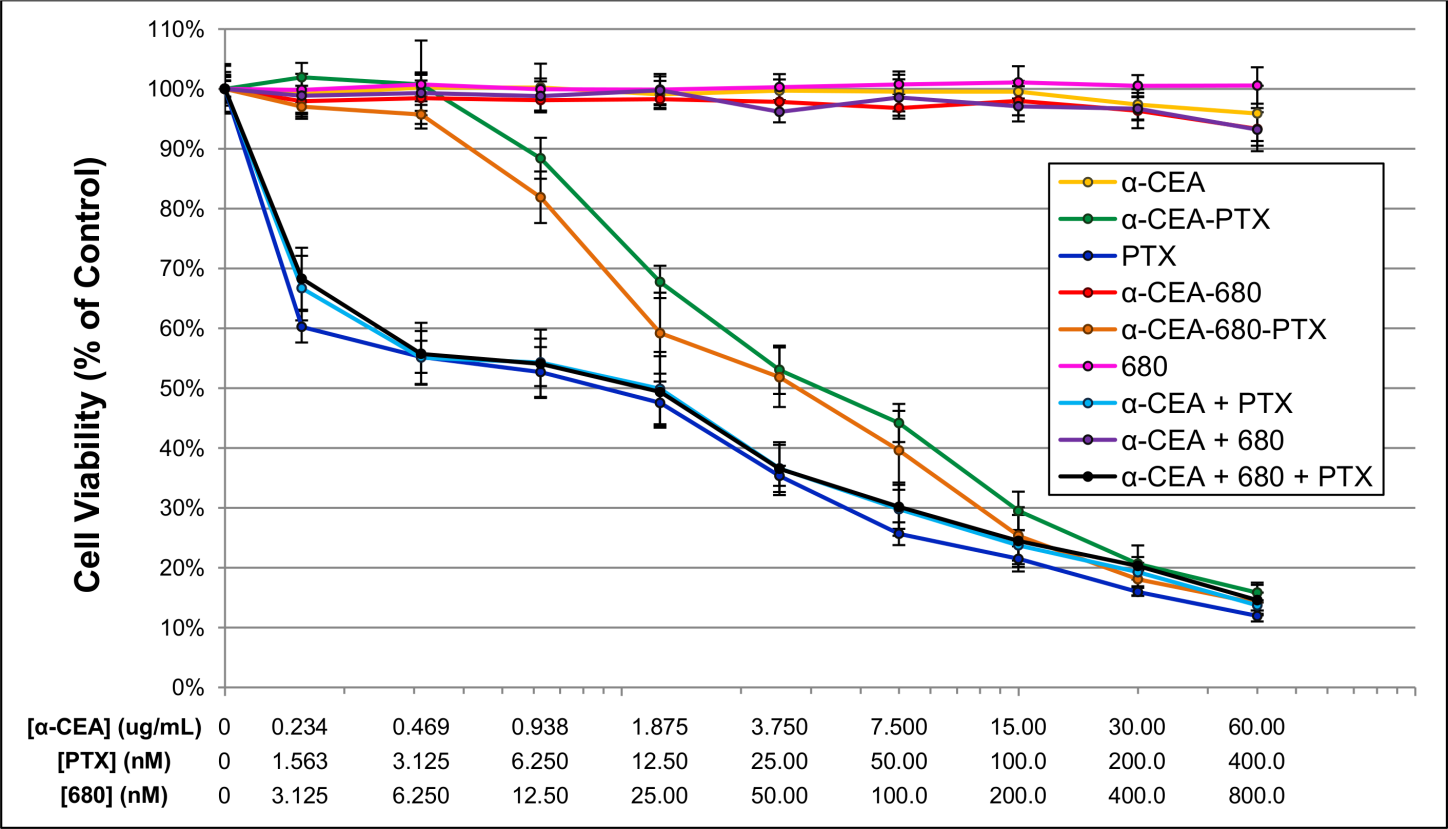
MTT Cytotoxic Assessment of Samples and Conjugates. BxPC-3 cells (5x10^3^) were plated in 96-well collagen-coated plates, and treated with a range of various test samples as noted in the figure. Connected dashes denote conjugates, and “+” indicates the addition of free, unconjugated compound (i.e. α-CEA-680 is the fluorescent antibody conjugate, where α-CEA + 680 indicates naked antibody plus free fluorescent dye). The sample concentration at each data point is plotted according to the three x-axes. For example, the data point on the far right for α-CEA-680-PTX is comprised of 60ug/mL of antibody, which corresponds to an effective paclitaxel concentration of 400nM, and an effective DyLight 680 concentration of 800nM. Samples are diluted 1:2 serially throughout the rest of the series. Samples that do not contain antibody, paclitaxel, or DyLight 680 are still standardized in this manner for graphical comparison (i.e. the far right data point for naked antibody, α-CEA, is comprised of a 60ug/mL solution and serially diluted 1:2 as before, and α-CEA-PTX starts at 60ug/mL, which corresponds to an effective paclitaxel concentration of 400nM, and is serially diluted identically.) After 24 hours, samples were removed and replaced with fresh media. Cells were allowed to grow for 72 additional hours and then stained with MTT according to the manufacturer’s instructions. The absorbance of each well was measured at 540nm on the Thermo Scientific VarioSkan Flash plate spectrophotometer. Edges of the plate were omitted. Percent viability was defined as the ratio of the A540 of test wells compared to the A540 of untreated control wells. Untreated controls received media and an appropriate vehicle depending on sample. Data points represent the average of three separate experiments performed in triplicate (n = 9 wells). Error bars denote standard error.

A subsequent major aim of this study was to demonstrate that the fluorescent ADC was cytotoxic to target cells. Using an *in vitro* MTT cell viability assay, BxPC-3 cells were treated with different modified CEA antibodies and compared to free and antibody-bound drug and fluorophore controls. As shown in the compiled dose-response curves (Fig 6), CEA antibody alone was not intrinsically cytotoxic. DyLight™ 680-4xPEG, or conjugates thereof, were also non-toxic. Only samples containing paclitaxel, whether free or bound to antibody, were shown to exhibit dose-dependent decreases in cell viability.

Using the dose response curves shown in Fig 6, IC_50_ values for each condition were estimated (Table 2). The IC_50_ of samples containing free paclitaxel (PTX, α-CEA + PTX, α-CEA + 680 + PTX) were approximately 3-fold lower than the IC_50_ of samples with paclitaxel bound to antibody (α-CEA-PTX and α-CEA-680-PTX). In comparing various conjugates, α-CEA-680-PTX was more potent than that of α-CEA-PTX at almost every concentration in the MTT assay (Fig 6). Furthermore, the estimated IC_50_ value for α-CEA-680-PTX was also 20% lower than α-CEA-PTX (Table 2). This difference is most likely due to the increased DAR of α-CEA-680-PTX, which happens to be 20% higher than α-CEA-PTX (Table 1).

**Table 2:** IC_50_ Estimations of Samples and Conjugates.

**Sample**	**Estimated IC**_**50**_ **(nM)**
α-CEA	NA*
α-CEA-PTX	37.99
PTX	9.84
α-CEA-680	NA*
α-CEA-680-PTX	30.13
680	NA*
α-CEA + PTX	11.89
α-CEA + 680	NA*
α-CEA + 680 + PTX	12.16

*Not applicable, value too large to compute

Previous reports have shown an increase in the effectiveness of paclitaxel when bound to antibody in other cytotoxic assays [56–59]. However, our results indicate that free paclitaxel was more cytotoxically potent *in vitro* than equimolar levels conjugated to an antibody. One possible explanation for these results is that free paclitaxel can more easily diffuse into cells *in vitro* compared to paclitaxel ADCs that must go through CEA binding, internalization, and degradation before eventual intracellular drug release. In addition, a majority of paclitaxel ADCs previously reported couple paclitaxel to antibodies which also exhibit some cytotoxic effects [56–59]. Although CEA is an effective target for immunofluorescent diagnostics (Figs 3–5) and visualization of tumors [66–81], we have demonstrated that CEA antibodies alone do not contribute to cytotoxicity (Fig 6), likely due to the fact that CEA is not necessary for cell survival [60, 61]. Further, drug-free antibody control samples (α-CEA-680, IgG-680) exhibited no decrease in cell survival even after prolonged incubation times (Fig 5).

**Fig 7:**
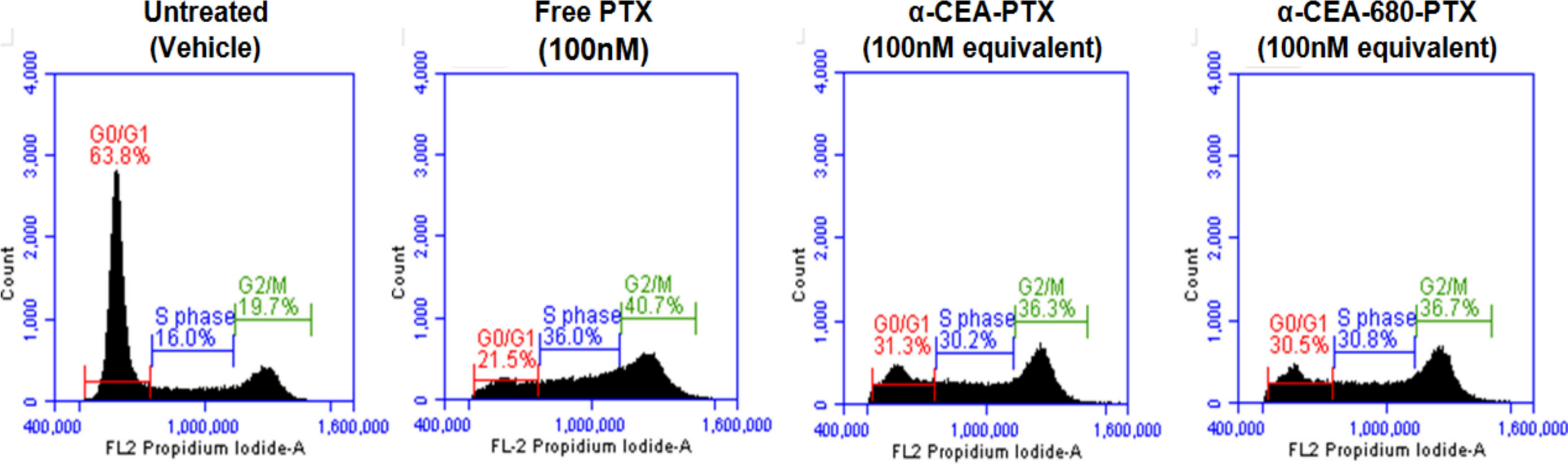
Flow Cytometry Cell Cycle Analysis. BxPC-3 cells were treated with vehicle control, 100nM free paclitaxel, 15ug/mL α-CEA-PTX, or 15ug/mL α-CEA-680-PTX (both at 100nM effective paclitaxel) for 24 hours. Cell cycle analysis was analyzed using the BD Cycletest™ Plus DNA Reagent Kit. All samples were gated identically, and 30,000–40,000 events were collected for each sample.

The molecular cytotoxic mechanism of free and antibody-conjugated paclitaxel was also assessed using the BD Cycletest™ Plus DNA ploidy staining kit for flow cytometric cell cycle analysis. Efficacy of α-CEA-PTX and α-CEA-680-PTX was also compared in the assay to further assess the effects of fluorescent dye conjugation on ADC functionality. As shown in Fig 7, both α-CEA-PTX and α-CEA-680-PTX antibody conjugate treatment resulted in a similar cell cycle arrest at G_2_/M phase, consistent with the established molecular mechanism of paclitaxel. Similar to our MTT cytotoxicity assay results, free paclitaxel was more efficient at arresting cells than equimolar levels of paclitaxel bound to an antibody. These results demonstrate the cytotoxic functionality of our fluorescent ADC and that fluorescent dye labeling did not significantly alter this activity.

### *In Vivo* Evaluation

**Fig 8:**
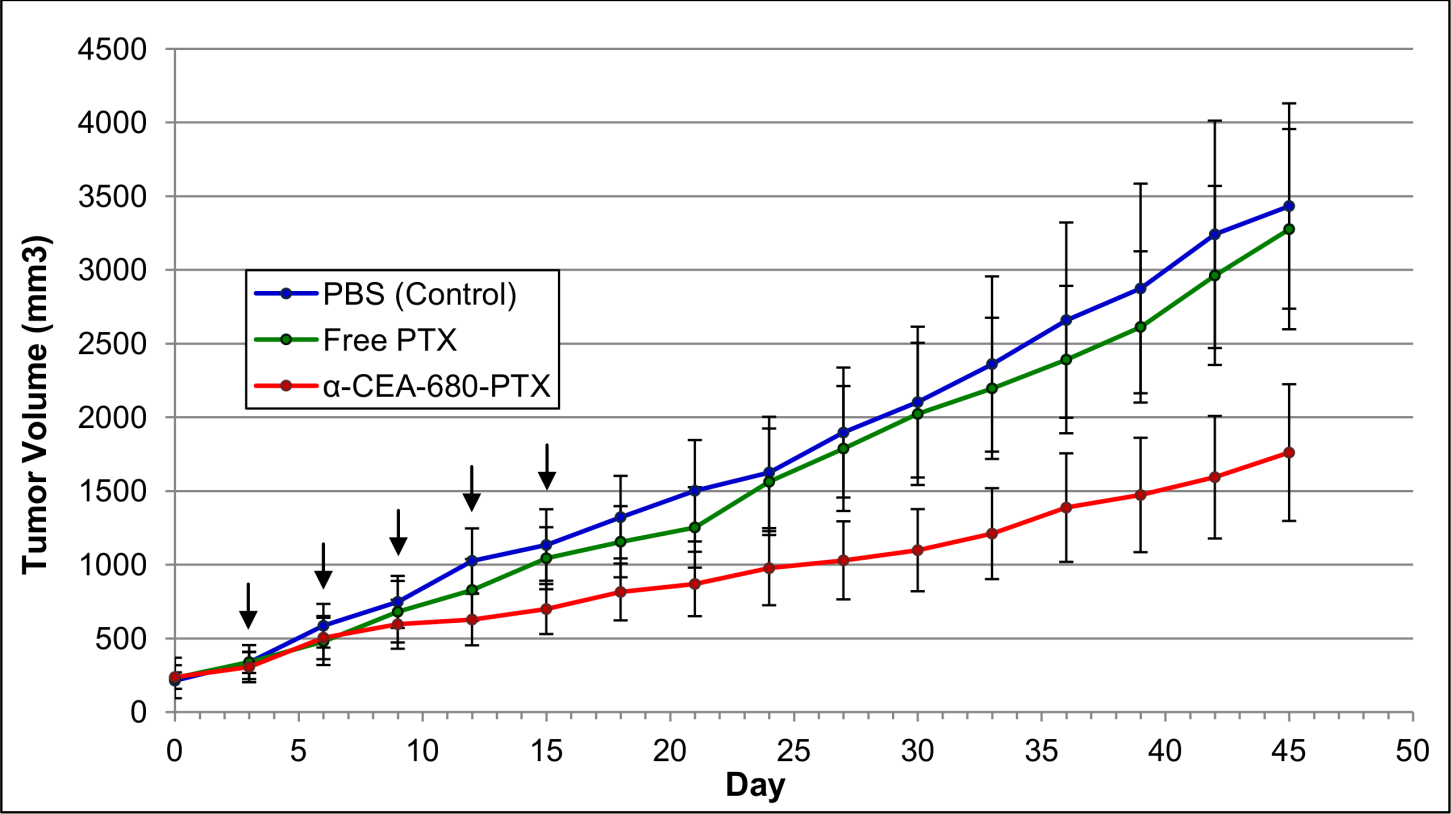
*In Vivo* Tumor Growth Inhibition. Male nude, athymic mice, 4 weeks of age, were subcutaneously inoculated with BxPC-3 cells (2x10^6^) embedded in 50:50 HBSS:Matrigel. When tumors were palpable (~1 week), mice were tagged and randomly separated into 3 groups. Mice in group 1 (n = 6) received PBS (vehicle). Group 2 (n = 6) received free paclitaxel (6.7uM) in PBS, while group 3 (n = 5) received equimolar amounts of paclitaxel conjugated to antibody (fluorescent ADC, 100ug α-CEA-680-PTX, 6.7uM effective paclitaxel). Each mouse received a total of five doses of the respective treatment, spaced 3 days apart, via retro-orbital injection. Arrows indicate treatment days (days 3, 6, 9, 12, and 15). Tumor size was measured every 3 days using digital calipers, and tumor volume was calculated with the modified ellipsoid formula (v = ½(length × width^2^)). Each data point represents average tumor volume (n = 5–6 mice). and error bars denote standard error.

To demonstrate specificity and cytotoxicity of the fluorescent ADC *in vivo*, a mouse tumor xenograft model was used. Nude, athymic mice were subcutaneously inoculated with BxPC-3 cells. When tumors were palpable, mice were separated randomly into 3 groups. Mice in group 1 (n = 6) were negative controls and received PBS (vehicle). Group 2 (n = 6) received free paclitaxel (6.7uM) in PBS, while group 3 (n = 5) received equimolar amounts of paclitaxel conjugated to antibody (100ug α-CEA-680-PTX, 6.7uM effective paclitaxel). Each mouse received a total of five doses of the respective treatment, spaced 3 days apart, via retro-orbital injection. Tumor growth was measured and calculated every 3 days throughout the study, and average tumor volume for each group data is plotted in Fig 8.

As shown from the tumor growth inhibition curves in Fig 8, clear differences in tumor volume are observed between α-CEA-680-PTX compared to both PBS and free PTX control groups over time. In addition, conjugating paclitaxel to a tumor-specific antibody appeared to increase its efficacy compared to equimolar levels of free paclitaxel, which is at an otherwise low, clinically irrelevant dose. However, when the average volume of each group on day 45 was compared with a one-way ANOVA, these differences were not found to be significantly different (*F*(2,14) = 1.803, *p* = 0.2011). Large variability was observed in tumor depth, shape, and volume across mice in all groups, and it is likely this was a major contributor to a lack of significant differences, particularly when considering the relatively low number of mice (n = 5–6 in each group) in this pilot study.

Based on our observations, α-CEA-680-PTX localizing to a BxPC-3 tumor *in vivo* may exhibit three potential outcomes; a) α-CEA-680-PTX localizes to the tumor site, but paclitaxel has already been hydrolyzed from the antibody en route due to linker lability (Fig 2); b) α-CEA-680-PTX localizes, is internalized with CEA, digested via lysosomal degradation, and the drug is released intracellularly; or c) α-CEA-680-PTX localizes, paclitaxel is hydrolyzed from the antibody before internalization occurs, released to the local extracellular environment, and passively diffuses into target cells.

Given these potential pathways, the biochemical design of our fluorescent ADC contains a number of areas for improving this efficacy. As alluded to earlier, it is clear that antibody-drug linker stability is a critical parameter for effective tumor growth inhibition, and Safavy et al. described an addition of an extra carbon spacer to this linker which increased physiological stability by at least 16-fold [83], a modification which would likely enhance *in vivo* effectiveness of our own conjugate. Furthermore, conjugating higher amounts of drug typically confers higher potency in ADCs, and using PEG itself as a linker with paclitaxel has been shown to yield soluble antibody conjugates with DARs at 10:1 and upwards [59]. The antibody itself also presents an opportunity for greater effectiveness. While CEA is an established target for diagnostic visualization of cancer, antibodies against the biomarker in this study did not confer any cytotoxic properties (Fig 6). Utilizing an antibody that is intrinsically therapeutic or targeted against vital receptors that normally bind ligands for growth factors, nutrients, or other survival signals would likely improve the effectiveness of this conjugate. Indeed, almost all ADCs in the clinical pipeline share this common feature [9, 10, 17]. Additionally, conjugating different drugs may increase the fluorescent ADC’s potency, solubility, and overall efficacy, and would be intriguing for subsequent experimentation.

**Fig 9:**
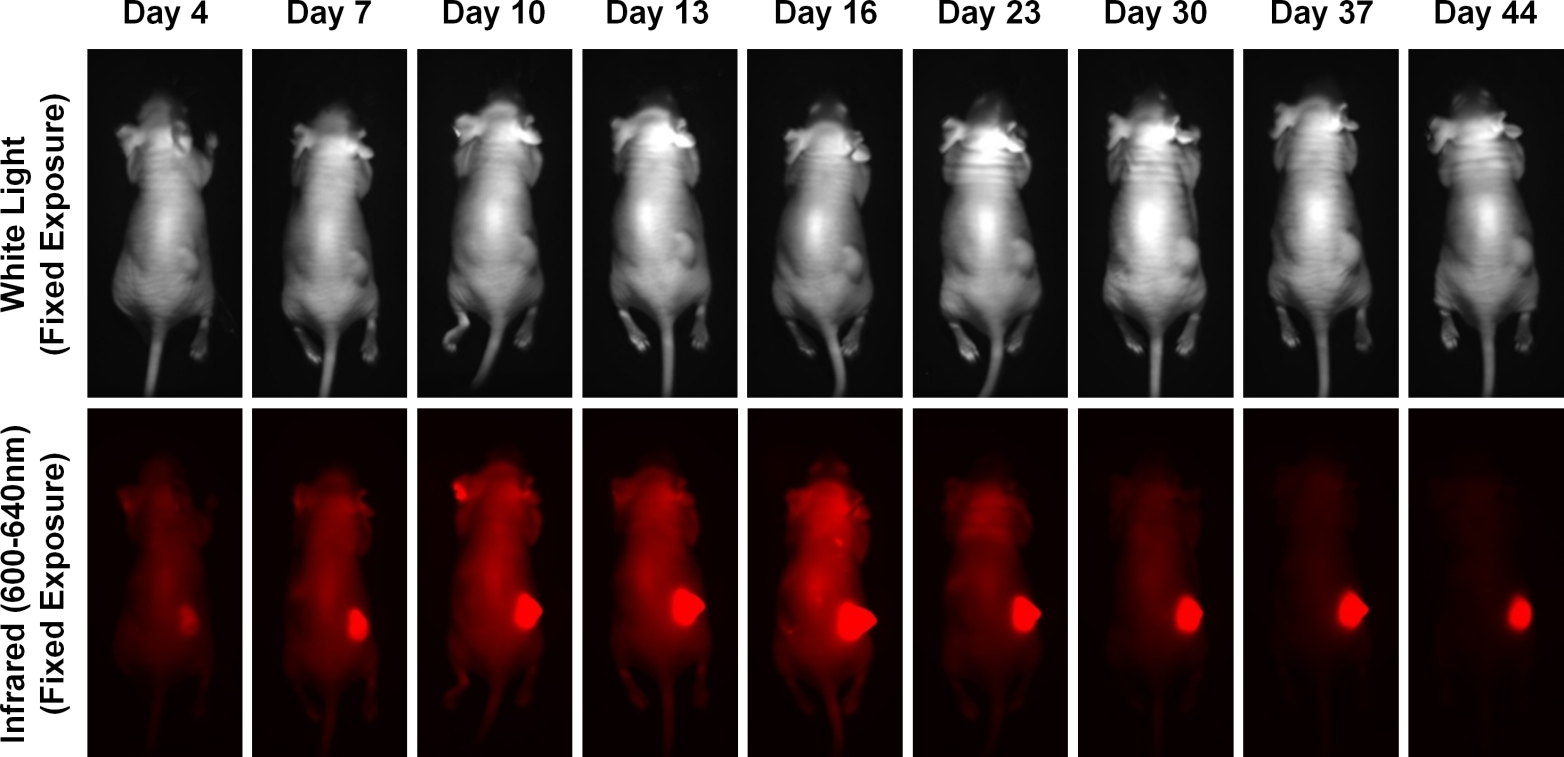
*In Vivo* Fluorescent Tumor Localization of α-CEA-680-PTX. Mice in group 3 were imaged 24 hours after every treatment infusion (i.e. treatment on days 3, 6, 9, 12, and 15, imaging on days 4, 7, 10, 13, and 16), and were also imaged once a week after all treatment had stopped (days 23, 30, 37, and 44). At each time point, mice were anesthetized as outlined in the methods section and imaged using the UVP iBox Explorer^2^ with attached BioLite Xe MultiSpectral Source. Images were obtained with a white light (control) and a NIR excitation filter (600-645nm), an emission filter of 720nm, a magnification of 0.17x, and fixed exposure times as indicated.

**Fig 10:**
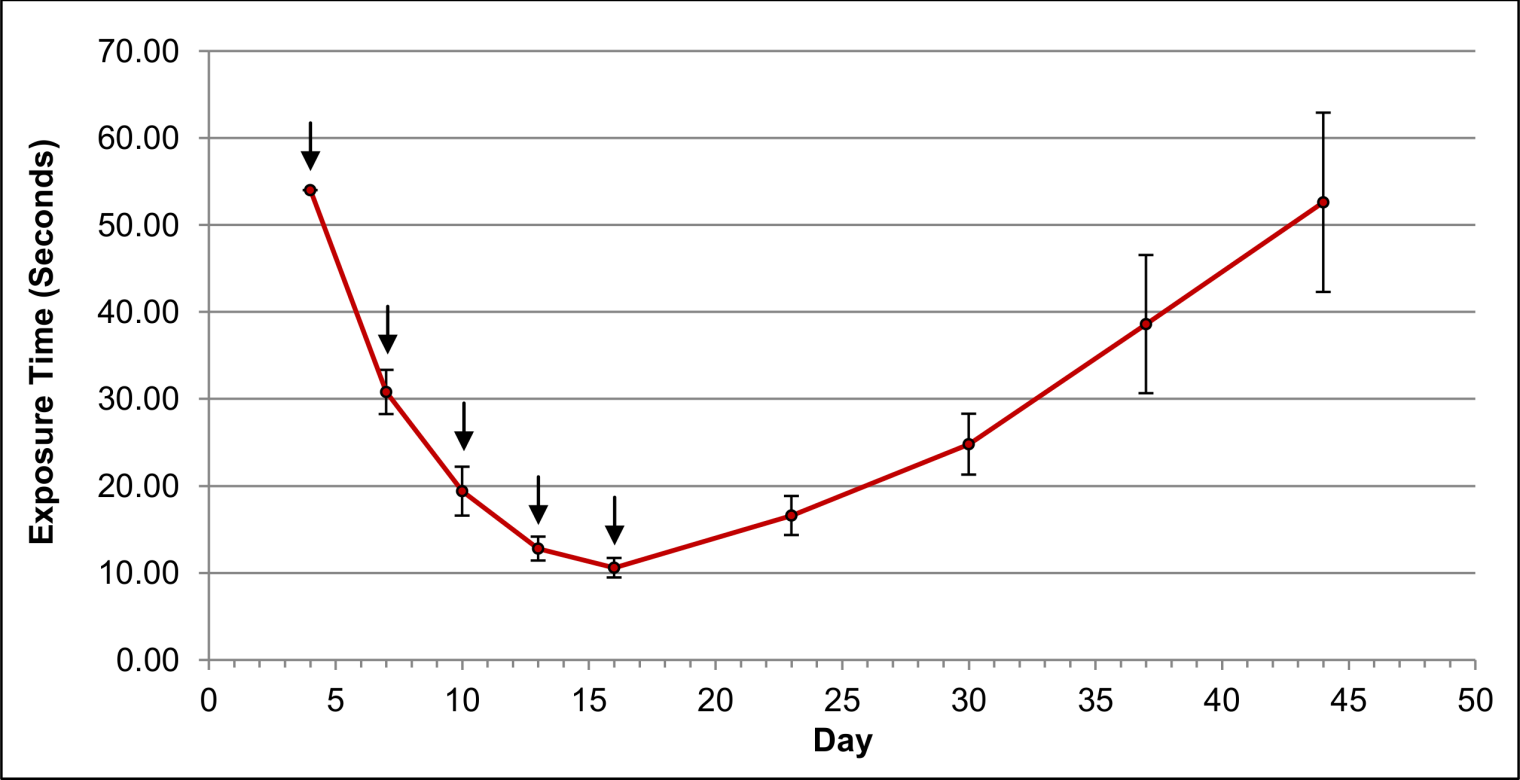
Automatic Exposure Times Throughout the Study. Mice in group 3 were imaged 24 hours after every treatment infusion (i.e. treatment on days 3, 6, 9, 12, and 15, imaging on days 4, 7, 10, 13, and 16), and were also imaged once a week after all treatment had stopped (days 23, 30, 37, and 44). At each time point, mice were anesthetized as outlined in the methods section and imaged using the UVP iBox Explorer^2^ with attached BioLite Xe MultiSpectral Source. A variable automatic exposure using the VisionWorks control software was obtained on the days indicated. Treatment infusion days are indicated by arrows (days 3, 6, 9, 12, and 15). Data points represent average automatic exposure times (n = 5 mice). Error bars denote standard error.

Mice in group 3 were also imaged 24 hours after each treatment infusion, followed by weekly imaging as described in the methods section. The fluorescent tumor localization images of introduced α-CEA-680-PTX are shown in Fig 9. Both excitation filter sets are depicted. Images were obtained at the time points indicated, with fixed exposure times where noted. Signal intensity and specificity are clearly illustrated, and are consistent with previous reports demonstrating *in vivo* CEA detection, particularly with fluorescently labeled antibodies [72–81]. In addition to fixed exposure times, automatic exposures were obtained for the 600-640nm excitation on the dorsal side of mice in group 3, and the average exposure time at different points in the study is plotted in Fig 10.

Images, as well as the exposure times obtained throughout the study, show strong tumor fluorescence and substantial signal stability still observed 4 weeks after α-CEA-680-PTX administration. While *in vitro* internalization kinetics of antibody-bound CEA were estimated in this particular system (Fig 5), the precise molecular composition of the remaining fluorescent entity is unknown, as well as how long the signal indefinitely persists *in vivo*. Based on our assessment of CEA internalization kinetics, particularly in BxPC-3 cells treated with α-CEA-680-PTX, it is likely that the signal longevity observed in mice is a combination of externally bound, intact antibody conjugate and internalized, digested conjugate with accumulated fluorophore inside apoptotic cells. Regardless, the signal decay was highly predictable after stopping treatment infusions with an estimated half-life of approximately 14 days (Fig 10). Ultimately, more accurate pharmacodynamic methods are needed to assess α-CEA-680-PTX half-life and tumor internalization. Replicating this experiment, followed by tumor excision and further characterization of the remaining conjugate and/or fluorophore would be highly interesting for further study.

Looking at Figs 9 and 10, it is clear that signal intensity increased with each treatment infusion, potentially indicating that the current dosing scheme was suboptimal and that this dose (100ug fluorescent ADC) was insufficient for complete tumor saturation. The variability observed in tumor size also suggests that the current dose may also only be appropriate for tumors of a certain volume or stage. Although our *in vivo* assessment of our dual-labeled ADCs showed high tumor localization specificity, our combined data suggest that an increased dose, administered earlier in the tumor growth cycle, and spaced further apart may have been more effective at significantly reducing tumor volume.

## Discussion

Therapeutic antibodies and ADCs continue to exhibit great promise as cancer treatment options and drug delivery systems. Radio-labeled or fluorescently-tagged antibodies have also demonstrated their effectiveness as valuable tools for cancer detection, diagnosis, and surgical guidance. Our study addresses the need to unify these concepts, presenting a simple and quick method for synthesizing a dual-labeled fluorescent antibody-drug conjugate. Using amine-reactive chemistry, we conjugated a monoclonal antibody specific for carcinoembryonic antigen (CEA) to either paclitaxel, a near-infrared fluorophore (DyLight™ 680-4xPEG), or both simultaneously. Furthermore, the use of a resin-based purification method enabled near complete removal of un-reacted components, resulting in highly pure constructs. Through various *in vitro* and *in vivo* analyses, we demonstrate feasibility of this bifunctional antibody conjugate as a theranostic agent.

While further study is needed to improve the pharmacokinetics, stability, and potency of this construct for optimal imaging and tumor growth inhibition, the technique reported here is an effective methodology for synthesizing a dual-labeled antibody for simultaneous molecular imaging and targeted therapy of cancer. The increasingly wide availability of monoclonal antibodies and recombinant protein technology, as well as the progressive nature of both drug and molecular imaging agent discovery, presents a significant opportunity to evaluate the diagnostic and therapeutic potential of a continually growing number of components. The method we present in this study is a highly cost effective and accessible tool utilizing readily available, off-the-shelf reagents, and can be employed to produce a vast variety of antibody or protein conjugates for extensive, high-throughput screening and pre-clinical evaluation. Further, this protocol can potentially decrease costs associated with *in vivo* imaging and ADC research, thereby increasing the accessibility of studying these strategies in preclinical cancer treatment and molecular diagnostics.

## Materials and Methods

### Antibodies

Mouse monoclonal antibody (IgG_1_ isotype, clone 1105) specific for carcinoembryonic antigen was obtained from Thermo Fisher Scientific (Product #MIC0101, Rockford, IL). This antibody was proprietarily generated by Thermo Fisher Scientific, in BALB/c mice immunized with purified CEA from pooled tumors. Isolated primary splenocytes were fused with P3X63-Ag8.653 mouse myeloma cell line (American Type Culture Collection, #CRL-1580, Manassas VA). Resulting mouse antibody was purified with immobilized, recombinant Protein A.

A mouse IgG_1_ isotype control was also utilized to prepare control conjugate samples. Isotype control was obtained from Thermo Fisher Scientific (Product # 02–6100). This antibody was proprietarily generated and purified from mouse myeloma IgG_1_ ascites induced by myeloma cells.

### Synthesis of Paclitaxel-NHS Ester (PTX-NHS)

Paclitaxel (TCI America, #P1632, Portland, OR) was derivatized into an amine-reactive succinate ester by the general procedure previously outlined in [51] with purity and expected mass verified with analysis on a Thermo Scientific LTQ XL™ Linear Ion Trap Mass Spectrometer.

### Synthesis, Purification, and Characterization of Conjugates

#### General procedure

Antibody conjugates were prepared and analyzed according to the general schematic shown in Fig 1. Mouse monoclonal antibody (anti-human CEA or non-binding IgG_1_ Isotype control) was buffer exchanged and processed with a 30kD PES concentrator (Thermo Fisher Scientific, #88502, Rockford, IL) to yield a 10mg/mL antibody solution in 0.05M sodium borate buffer, pH 8.5. Paclitaxel-NHS and DyLight™ 680-4xPEG-NHS (Thermo Fisher Scientific, #46603, Rockford, IL) stocks were prepared in DMSO. Where appropriate for each reaction, either a 5-fold molar excess of PTX-NHS, a 3-fold molar excess of the DyLight™ 680-4xPEG-NHS, or both simultaneously, were reacted with 1mg of the processed CEA antibody for a final volume of 100uL with 5% DMSO co-solvent. Reactions were incubated for 1 hour at room temperature with gentle vortexing every 10 minutes. Reactions were neutralized in PBS and purified with 3x100uL incubations with the resin provided in the Pierce™ Dye Removal Column kit (Thermo Fisher Scientific, #22858, Rockford, IL). Purified samples were then dialyzed extensively in PBS overnight using a 20kD Slide-A-Lyzer™ G2 Dialysis Cassette (Thermo Fisher Scientific, #87734, Rockford, IL) to remove residual solvent and preservatives. Protein concentration and fluorophore:antibody ratio (FAR), where applicable, were determined using spectrophotometric absorbance on an Agilent Cary 300 UV-Vis spectrophotometer. Drug:antibody ratio (DAR), where applicable, was determined by hydrolyzing the antibody-drug ester bond linkage and quantifying extracted paclitaxel. Triplicate samples (5ug) of purified conjugates were hydrolyzed in 50uL 0.2M carbonate-bicarbonate buffer, pH 9.4 for 6 hours at room temperature. Samples were then adjusted to 30% ACN, and liberated paclitaxel was extracted with C18 Spin Columns (Thermo Fisher Scientific, #89870, Rockford, IL). Bound paclitaxel was eluted in 100% ACN and analyzed using an Agilent 1100 HPLC with a Phenomenex Gemini® 5 μm C18 column (#00F-4435-B0, Torrance, CA) and a mobile phase linear gradient of 50% to 100% ACN in water over 25 minutes. Instrument control and chromatogram analysis were completed using OpenLab CDS ChemStation Edition software (vA01.04). Extracted paclitaxel from conjugates was quantified by correlating peak areas to known, triplicate amounts of free paclitaxel that were hydrolyzed, extracted and analyzed with the same procedure.

#### Verification of dye removal column purification method

To assess and verify removal of un-reacted components (PTX-NHS and DyLight™ 680-4xPEG-NHS) using the Pierce™ Dye Removal Column resin, mock conjugation reactions were prepared as described in the methods section, albeit in the absence of antibody. These reactions were purified with the resin and analyzed on the HPLC system described earlier. For PTX-NHS removal, un-purified and purified samples were injected onto the Gemini® column with a mobile phase linear gradient of 50% to 100% ACN in water over 25 minutes. Known amounts of PTX-NHS were also serially diluted and analyzed in triplicate to approximate the amount of remaining PTX-NHS after purification. For DyLight™ 680-4xPEG-NHS removal, samples were injected onto the same column as described earlier, but with a mobile phase linear gradient of 0% to 100% ACN in water over 25 minutes, with both solvents also containing 0.1%Trifluoroacetic acid. Known amounts of DyLight™ 680-4xPEG-NHS were also analyzed with this method for removal estimation. Instrument control and chromatogram analysis were completed as previously described.

#### Antibody-drug linker stability evaluation

To estimate physiological stability of the antibody-drug linker, 2.5ug of the fluorescent ADC (α-CEA-680-PTX) was added to 50uL of PBS and incubated at 37°C. Hydrolyzed control samples were also prepared, comprised of equimolar amounts of naked CEA antibody and free paclitaxel with respect to test samples. Samples were taken in triplicate at T = 0, 2, 4, 8, 24 and 48 hours, and free paclitaxel was extracted and analyzed with C18 spin columns as previously described. Degree of hydrolysis at each time point (n = 3) was defined as the peak area ratio of extracted paclitaxel from test samples compared to hydrolyzed controls.

### *In Vitro* Specificity Evaluation

#### Immunofluorescent staining and analysis

BxPC-3, MCF-7, HeLa and HepG2 cell lines (American Type Culture Collection, Manassas, VA) were cultured in RPMI-1640 + 10% FBS (Thermo Fisher Scientific, #11875–085, Grand Island, NY) at 37°C, 5% CO_2_, and harvested with 0.25%Trypsin-EDTA (Thermo Fisher Scientific, #25200–056, Grand Island, NY). Cells were plated in 96-well collagen I-coated plates (Corning Incorporated, #356700, Corning, NY) at 1x10^4^ cells/well and allowed to recover for 24 hours. Cells were then fixed with pre-warmed PBS + 4% paraformaldehyde and blocked with PBS + 0.3% BSA. Conjugates (IgG-680, IgG-680-PTX, α-CEA-680, and α-CEA-680-PTX) were serially diluted 1:2 (ranging from 0-60ug/mL) in PBS + 0.3% BSA, 1.62uM Hoechst 33342 dye (Thermo Fisher Scientific, #62249, Rockford, IL) and incubated in appropriate wells for 1 hour at room temperature. Plates were then washed twice with both PBS + 0.05% Tween 20, and PBS. Average fluorescent intensity was analyzed using the Compartmental Analysis v2 BioApplication on the Thermo Scientific Cellomics ArrayScan VTI HCS Reader. Average fluorescent intensity was measured as the mean intensity of 3000 identified cells. All plates were read using the same method parameters. Instrument control, analysis and quantification was performed using the vHCS Scan software (v6.1.2). Images were captured at 20X magnification, and enhanced using the vHCS View software (v1.4.6),Microsoft PowerPoint 2007 (v12.0.6665.5003), and AutoDesk Pixlr Editor (v6.7). All images were enhanced identically. Edges of the plate were omitted.

#### Flow cytometry staining

BxPC-3, MCF-7, HeLa and HepG2 cells were cultured and harvested as previously described. Cells (1x10^6^) were equilibrated in cold PBS + 5% FCS, and incubated with a 10ug/mL solution of either IgG-680, IgG-680-PTX, α-CEA-680, or α-CEA-680-PTX for 1 hour. Cells were then washed and re-suspended with PBS + 5% FCS. Analysis was completed using the Becton Dickson Accuri C6 Flow Cytometer and CSampler Workspace software (v1.0.264.21). For each histogram, 50,000 events were collected.

#### Immunofluorescent CEA internalization assay

BxPC-3 cells were cultured and harvested as previously described, and seeded into 96-well collagen I-coated plates at 1x10^4^ cells/well and allowed to recover for 24 hours. Media was then removed, and cells were treated with 46ng/mL free DyLight™ 680-4xPEG, or 2ug/mL of IgG-680, IgG-680-PTX, α-CEA-680, or α-CEA-680-PTX, (all at 46ng/mL effective DyLight™ 680-4xPEG) diluted in complete RPMI-1640. Cells were incubated with samples for the indicated time points at 37°C, 5% CO_2_, after which the plates were then washed three times with PBS. Plates were then fixed with PBS + 4% paraformaldehyde, and nuclei were stained with 1.62uM Hoechst 33342 dye in PBS. Plates were washed an additional three times with PBS, and analyzed using a Thermo Scientific Cellomics ArrayScan VTI HCS Reader. Quantification of CEA internalization was assessed through measurement of mean internalized spots per cell (n = 10,000 cells for each time point) using the Spot Detector v2 Image Analysis BioApplication on the ArrayScan VTI HCS Reader. Instrument control was performed with the vHCS Scan software (v6.1.2), All plates were read using the same method parameters. Images were captured at 20X magnification, and enhanced using the vHCS View software (v1.4.6), Microsoft PowerPoint 2007 (v12.0.6665.5003), and AutoDesk Pixlr Editor (v6.7). All images were enhanced identically.

### *In Vitro* Cytotoxic Evaluation

#### MTT growth inhibition assay

Cytotoxic activity *in vitro* was assessed using an MTT (Thermo Fisher Scientific, #M6494, Grand Island, NY) cell viability assay. BxPC-3 cells were cultured and harvested as previously described, and were plated in 96-well collagen I-coated plates at 5x10^3^ cells/well and allowed to recover for 24 hours. Media was removed, and replaced with serially diluted (1:2 in complete RPMI-1640) test samples, ranging from 0-400nM free or effective paclitaxel, 0-60ug/mL antibody/conjugate, or 0-800nM free or effective DyLight™ 680-4xPEG, where applicable. After 24 hours, samples were removed and replaced with fresh media. Cells were allowed to grow for 72 additional hours, after which the cells were stained with MTT according to the manufacturer’s instructions. Liquid was carefully aspirated from each well, and replaced with 100uL DMSO to solubilize produced formazan. The absorbance of each well was measured at 540nm on the Thermo Scientific VarioSkan Flash plate spectrophotometer. Edges of the plate were omitted. Percent viability was defined as the ratio of the A540 of test wells compared to the A540 of untreated control wells. Untreated controls received media + vehicle depending on the sample. Each data point represents the average viability of three separate experiments each with triplicate wells (n = 9 wells). IC_50_ values were estimated using logarithmic regression analysis in Microsoft Excel.

#### Flow cytometry cell cycle analysis

Cell cycle analysis was performed using the Becton Dickson BD Cycletest™ Plus DNA Reagent Kit (BD, #340242, San Jose, CA). BxPC-3 cells were seeded equally into T25 flasks, and allowed to recover for 24 hours. Flasks were then treated with vehicle control, 100nM free paclitaxel, 15ug/mL α-CEA-PTX, or 15ug/mL α-CEA-680-PTX (both at 100nM effective paclitaxel) for 24 hours. Cells were then harvested with Trypsin-EDTA, stained according to the kit instructions, and analyzed via flow cytometry as previously detailed. All samples were gated and analyzed identically per the manufacturer/template instructions, with the cell cycle histograms containing approximately 30,000–40,000 events.

### *In Vivo* Tumor Localization and Growth Inhibition

#### Animals

Male nude, athymic mice, 4 weeks of age (Taconic Biosciences, #NCRNU-M, Hudson, NY) were group-housed (5 per cage) in a sterile microisolator cage system (Tecniplast Smart Flow). Mice were given irradiated rodent diet (Envigo Labs) and autoclaved water ad libitum. Each cage was provided with an autoclaved mouse hut and shredded paper for nesting and thermoregulation. Animals were checked daily by the animal care staff and/or the researcher for any adverse events throughout the course of the study. Animals that were considered ill or showing pain and/or distress were examined by the veterinarian for pain management determination. When using anesthesia, animals were observed every 30 minutes until recovery from anesthesia. Two mice (one each in experimental groups 1 and 3) died prior to the experimental endpoint. No autopsy was performed, and air embolism after treatment infusion was the likely cause of death. The duration of this study was planned for a total of 60 days, however several animals became increasingly moribund, with two mice (one each in experimental groups 1 and 2) having mobility issues with their hind legs. It was determined to end the study early (45 days total) to avoid further pain and distress of the animals. Mice were euthanized via CO2 (EZ- Flow system) asphyxiation followed by cervical dislocation. Humane use of animals was performed in this study according to the guidelines for the care and use of laboratory animals and with the rules formulated under the Animal Welfare Act by the U.S. Department of Agriculture. The protocol was approved by the IACUC Biological Resource Committee of the University of Illinois, College of Medicine at Rockford, IL and performed at a facility accredited by AAALAC and USDA.

#### Tumor induction

After a one week acclimatization period, mice were subcutaneously inoculated with 2x10^6^ BxPC-3 cells embedded in 100uL of 50:50 HBSS:Matrigel (Corning Incorporated, #354262, Corning NY). When tumors were palpable (~1 week), the mice were tagged and separated randomly into 3 groups, assuring that each group had approximately equal average starting tumor volumes. This was defined as “Day 0”.

#### Treatment

Mice were separated into one control group and two treatment groups, consisting of 5–6 mice per group. Mice in group 1 (n = 6) were negative controls and received PBS + 0.13% DMSO (vehicle). Group 2 (n = 6) received free paclitaxel (6.7uM) in PBS + 0.13% DMSO, while group 3 (n = 5) received equimolar amounts of paclitaxel conjugated to antibody (100ug α-CEA-680-PTX, 6.7uM effective paclitaxel in + 0.13% DMSO). Each mouse received a total of five 100uL doses of the respective treatment by retro-orbital injection, with each administered on days 3, 6, 9, 12, and 15. All mice were anesthetized with 100mg/kg ketamine and 10mg/kg xylazine given intraperitoneally prior to all retro-orbital infusions.

#### Tumor growth monitoring

Tumor size was monitored every 3 days for the entire duration of the study using digital calipers. The modified ellipsoid formula was used to calculate tumor volume (v = ½(length × width^2^)).

#### Tumor imaging

Mice in group 3 were imaged 24 hours after every treatment (Days 4, 7, 10, 13, and 16), and weekly thereafter (Days 23, 30, 37, and 44). Mice were anesthetized as outlined earlier, and imaged using the UVP iBox Explorer^2^ with attached BioLite Xe MultiSpectral Source. Instrument control and image acquisition were performed using the UVP VisionWorks LS Acquisition and Analysis software (v8.0). Images were obtained with a white light (control) and NIR excitation filter (600-645nm), an emission filter of 720nm, and magnification of 0.17x. Exposure times for white light were approximately 30 seconds, while NIR was exposed at both a constant 54 seconds, as well as a variable automatic exposure using the VisionWorks control software. All other parameters were identical, and images were prepared for final presentation with Microsoft Office Picture Manager (v12.0.6413.1000), and AutoDesk Pixlr Editor (v6.7).

### Statistical Analysis

All graphical analysis was completed using Excel. Statistical analysis was performed with GraphPad Prism 6 (v. 6.07) statistics calculator. Where appropriate, data groups were statistically compared using a one-way ANOVA test. An alpha level of 0.05 was considered significant.

### Graphics

All artwork and graphics are original creations, assembled and modified using clip art, stock photos, and royalty-free images purchased from 123rf.com. Enhancements and arrangements were prepared using Pixlr Editor, PowerPoint, and Excel.

## References

[1] Dimitrov DS, Marks JD. Therapeutic antibodies: current state and future trends–is a paradigm change coming soon? Methods in Molecular Biology. 2009; 525:1–27 10.1007/978-1-59745-554-1_1 19252861 PMC3402212

[2] Dykstra M. Biological electron microscopy: theory, techniques, and troubleshooting New York and London: Plenum; 1992 pp 309–310.

[3] Wu A, Olafsen T. Antibodies for molecular imaging of cancer. The Cancer Journal. 2008;14(3):191–197. 10.1097/PPO.0b013e31817b07ae 18536559

[4] Rao J, Dragulescu-Andrasi A, Yao H. Fluorescence imaging in vivo: recent advances. Current Opinion in Biotechnology. 2007;18(1):17–25. 17234399 10.1016/j.copbio.2007.01.003

[5] Wu A, Senter P. Arming antibodies: prospects and challenges for immunoconjugates. Nat Biotechnol. 2005;23(9):1137–1146. 16151407 10.1038/nbt1141

[6] Polakis P. Arming antibodies for cancer therapy. Current Opinion in Pharmacology. 2005;5(4):382–387. 15951239 10.1016/j.coph.2005.04.008

[7] Carter P, Senter P. Antibody-drug conjugates for cancer therapy. The Cancer Journal. 2008;14(3):154–169. 10.1097/PPO.0b013e318172d704 18536555

[8] Kovtun Y, Goldmacher V. Cell killing by antibody–drug conjugates. Cancer Letters. 2007;255(2):232–240. 17553616 10.1016/j.canlet.2007.04.010

[9] Sassoon I, Blanc V. Antibody-drug conjugate (ADC) clinical pipeline: a review. Methods in Molecular Biology. 2013;1045:1–27. 10.1007/978-1-62703-541-5_1 23913138

[10] Mullard A. Maturing antibody–drug conjugate pipeline hits 30. Nat Rev Drug Discov. 2013;12(6):483–483. 10.1038/nrd4009 23629491

[11] Liu JK. The history of monoclonal antibody development–Progress, remaining challenges and future innovations. Annals of Medicine and Surgery. 2014 12 31;3(4):113–6. 10.1016/j.amsu.2014.09.001 25568796 PMC4284445

[12] Boutureira O, Bernardes GJ. Advances in chemical protein modification. Chemical Reviews. 2015 2 20;115(5):2174–95. 10.1021/cr500399p 25700113

[13] Chalker JM, Bernardes GJ, Lin YA, Davis BG. Chemical modification of proteins at cysteine: opportunities in chemistry and biology. Chemistry–An Asian Journal. 2009 5 4;4(5):630–40. 10.1002/asia.200800427 19235822

[14] Hermanson GT. Bioconjugate techniques Academic press; 2013 7 25.

[15] Jain N, Smith SW, Ghone S, Tomczuk B. Current ADC linker chemistry. Pharmaceutical research. 2015 11 1;32(11):3526–40. 10.1007/s11095-015-1657-7 25759187 PMC4596905

[16] Lyon RP, Meyer DL, Setter JR, Senter PD. 6 Conjugation of Anticancer Drugs Through Endogenous Monoclonal Antibody Cysteine Residues. Methods in enzymology. 2012 1 1;502:123 10.1016/B978-0-12-416039-2.00006-9 22208984

[17] Feng Y, Zhu Z, Chen W, Prabakaran P, Lin K, Dimitrov DS. Conjugates of small molecule drugs with antibodies and other proteins. Biomedicines. 2014 1 24;2(1):1–3. 28548057 10.3390/biomedicines2010001 PMC5423484

[18] Francisco JA, Cerveny CG, Meyer DL, Mixan BJ, Klussman K, Chace DF, Rejniak SX, Gordon KA, DeBlanc R, Toki BE, Law CL. cAC10-vcMMAE, an anti-CD30–monomethyl auristatin E conjugate with potent and selective antitumor activity. Blood. 2003 8 15;102(4):1458–65. 12714494 10.1182/blood-2003-01-0039

[19] Katz J, Janik JE, Younes A. Brentuximab vedotin (SGN-35). Clinical Cancer Research. 2011 10 15;17(20):6428–36. 10.1158/1078-0432.CCR-11-0488 22003070

[20] Naumovski L, Junutula JR. Glembatumumab vedotin, a conjugate of an anti-glycoprotein non-metastatic melanoma protein B mAb and monomethyl auristatin E for the treatment of melanoma and breast cancer. Current opinion in molecular therapeutics. 2010 4;12(2):248–57. 20373269

[21] Yu SF, Zheng B, Go M, Lau J, Spencer S, Raab H, Soriano R, Jhunjhunwala S, Cohen R, Caruso M, Polakis P. A Novel Anti-CD22 Anthracycline-Based Antibody–Drug Conjugate (ADC) That Overcomes Resistance to Auristatin-Based ADCs. Clinical Cancer Research. 2015 7 15;21(14):3298–306. 10.1158/1078-0432.CCR-14-2035 25840969

[22] Ma D, Hopf CE, Malewicz AD, Donovan GP, Senter PD, Goeckeler WF, Maddon PJ, Olson WC. Potent antitumor activity of an auristatin-conjugated, fully human monoclonal antibody to prostate-specific membrane antigen. Clinical Cancer Research. 2006 4 15;12(8):2591–6. 16638870 10.1158/1078-0432.CCR-05-2107

[23] Phillips GD, Li G, Dugger DL, Crocker LM, Parsons KL, Mai E, Blättler WA, Lambert JM, Chari RV, Lutz RJ, Wong WL. Targeting HER2-positive breast cancer with trastuzumab-DM1, an antibody–cytotoxic drug conjugate. Cancer research. 2008 11 15;68(22):9280–90. 10.1158/0008-5472.CAN-08-1776 19010901

[24] Sapra P, Stein R, Pickett J, Qu Z, Govindan SV, Cardillo TM, Hansen HJ, Horak ID, Griffiths GL, Goldenberg DM. Anti-CD74 antibody-doxorubicin conjugate, IMMU-110, in a human multiple myeloma xenograft and in monkeys. Clinical Cancer Research. 2005 7 15;11(14):5257–64. 16033844 10.1158/1078-0432.CCR-05-0204

[25] DiJoseph JF, Armellino DC, Boghaert ER, Khandke K, Dougher MM, Sridharan L, Kunz A, Hamann PR, Gorovits B, Udata C, Moran JK. Antibody-targeted chemotherapy with CMC-544: a CD22-targeted immunoconjugate of calicheamicin for the treatment of B-lymphoid malignancies. Blood. 2004 3 1;103(5):1807–14. 14615373 10.1182/blood-2003-07-2466

[26] Ikeda H, Hideshima T, Fulciniti M, Lutz RJ, Yasui H, Okawa Y, Kiziltepe T, Vallet S, Pozzi S, Santo L, Perrone G. The monoclonal antibody nBT062 conjugated to cytotoxic Maytansinoids has selective cytotoxicity against CD138-positive multiple myeloma cells in vitro and in vivo. Clinical Cancer Research. 2009 6 15;15(12):4028–37. 10.1158/1078-0432.CCR-08-2867 19509164

[27] Hong EE, Erickson H, Lutz RJ, Whiteman KR, Jones G, Kovtun Y, Blanc V, Lambert JM. Design of Coltuximab Ravtansine, a CD19-Targeting Antibody–Drug Conjugate (ADC) for the Treatment of B-Cell Malignancies: Structure–Activity Relationships and Preclinical Evaluation. Molecular pharmaceutics. 2015 4 27;12(6):1703–16. 10.1021/acs.molpharmaceut.5b00175 25856201

[28] Whiteman KR, Johnson HA, Mayo MF, Audette CA, Carrigan CN, LaBelle A, Zukerberg L, Lambert JM, Lutz RJ. Lorvotuzumab mertansine, a CD56-targeting antibody-drug conjugate with potent antitumor activity against small cell lung cancer in human xenograft models. InMAbs 2014 3 1 (Vol. 6, No. 2, pp. 556–566). Taylor & Francis. 10.4161/mabs.27756 PMC3984343 24492307

[29] Flygare John A., Pillow Thomas H., and Aristoff Paul. "Antibody‐drug conjugates for the treatment of cancer." Chemical biology & drug design 81 1 (2013): 113–121. 23253133 10.1111/cbdd.12085

[30] Hamblett KJ, Senter PD, Chace DF, Sun MM, Lenox J, Cerveny CG, Kissler KM, Bernhardt SX, Kopcha AK, Zabinski RF, Meyer DL. Effects of drug loading on the antitumor activity of a monoclonal antibody drug conjugate. Clinical Cancer Research. 2004 10 15;10(20):7063–70. 15501986 10.1158/1078-0432.CCR-04-0789

[31] Strop P, Liu SH, Dorywalska M, Delaria K, Dushin RG, Tran TT, Ho WH, Farias S, Casas MG, Abdiche Y, Zhou D. Location matters: site of conjugation modulates stability and pharmacokinetics of antibody drug conjugates. Chemistry & biology. 2013 2 21;20(2):161–7. 23438745 10.1016/j.chembiol.2013.01.010

[32] Junutula JR, Raab H, Clark S, Bhakta S, Leipold DD, Weir S, Chen Y, Simpson M, Tsai SP, Dennis MS, Lu Y. Site-specific conjugation of a cytotoxic drug to an antibody improves the therapeutic index. Nature biotechnology. 2008 8 1;26(8):925–32. 10.1038/nbt.1480 18641636

[33] Panowski S, Bhakta S, Raab H, Polakis P, Junutula JR. Site-specific antibody drug conjugates for cancer therapy. InMAbs 2014 1 1 (Vol. 6, No. 1, pp. 34–45). Taylor & Francis. 10.4161/mabs.27022 PMC3929453 24423619

[34] McDonagh CF, Turcott E, Westendorf L, Webster JB, Alley SC, Kim K, Andreyka J, Stone I, Hamblett KJ, Francisco JA, Carter P. Engineered antibody–drug conjugates with defined sites and stoichiometries of drug attachment. Protein Engineering Design and Selection. 2006 7 1;19(7):299–307. 10.1093/protein/gzl013 16644914

[35] Agarwal P, Bertozzi CR. Site-specific antibody–drug conjugates: the nexus of bioorthogonal chemistry, protein engineering, and drug development. Bioconjugate chemistry. 2015 1 30;26(2):176–92. 10.1021/bc5004982 25494884 PMC4335810

[36] Junutula JR, Flagella KM, Graham RA, Parsons KL, Ha E, Raab H, Bhakta S, Nguyen T, Dugger DL, Li G, Mai E. Engineered thio-trastuzumab-DM1 conjugate with an improved therapeutic index to target human epidermal growth factor receptor 2–positive breast cancer. Clinical Cancer Research. 2010 10 1;16(19):4769–78. 10.1158/1078-0432.CCR-10-0987 20805300

[37] Hofer T, Skeffington LR, Chapman CM, Rader C. Molecularly defined antibody conjugation through a selenocysteine interface. Biochemistry. 2009 11 17;48(50):12047–57. 10.1021/bi901744t 19894757 PMC2825887

[38] Axup JY, Bajjuri KM, Ritland M, Hutchins BM, Kim CH, Kazane SA, Halder R, Forsyth JS, Santidrian AF, Stafin K, Lu Y. Synthesis of site-specific antibody-drug conjugates using unnatural amino acids. Proceedings of the National Academy of Sciences. 2012 10 2;109(40):16101–6. 10.1073/pnas.1211023109 PMC3479532 22988081

[39] Boeggeman E, Ramakrishnan B, Pasek M, Manzoni M, Puri A, Loomis KH, Waybright TJ, Qasba PK. Site-specific conjugation of fluoroprobes to the remodeled Fc N-glycans of monoclonal antibodies using mutant glycosyltransferases: application for cell surface antigen detection. Bioconjugate chemistry. 2009 5 8;20(6):1228–36. 10.1021/bc900103p 19425533 PMC3464487

[40] Jeger S, Zimmermann K, Blanc A, Grünberg J, Honer M, Hunziker P, Struthers H, Schibli R. Site‐Specific and Stoichiometric Modification of Antibodies by Bacterial Transglutaminase. Angewandte Chemie International Edition. 2010 12 17;49(51):9995–7. 21110357 10.1002/anie.201004243

[41] Rabuka D, Rush JS, Wu P, Bertozzi CR. Site-specific chemical protein conjugation using genetically encoded aldehyde tags. Nature protocols. 2012 6 1;7(6):1052–67. 10.1038/nprot.2012.045 22576105 PMC3498491

[42] Behrens C, Liu B. Methods for site-specific drug conjugation to antibodies. mAbs. 2013;6(1):46–53. 10.4161/mabs.26632 PMC3929454 24135651

[43] Maruani A, Smith M, Miranda E, Chester K, Chudasama V, Caddick S. A plug-and-play approach to antibody-based therapeutics via a chemoselective dual click strategy. Nature Communications. 2015;6:6645 10.1038/ncomms7645 25824906 PMC4389247

[44] Schumacher FF, Nunes JP, Maruani A, Chudasama V, Smith ME, Chester KA, Baker JR, Caddick S. Next generation maleimides enable the controlled assembly of antibody–drug conjugates via native disulfide bond bridging. Organic & biomolecular chemistry. 2014;12(37):7261–9. 25103319 10.1039/c4ob01550a PMC4159697

[45] Nunes JP, Morais M, Vassileva V, Robinson E, Rajkumar VS, Smith ME, Pedley RB, Caddick S, Baker JR, Chudasama V. Functional native disulfide bridging enables delivery of a potent, stable and targeted antibody–drug conjugate (ADC). Chemical Communications. 2015;51(53):10624–7. 10.1039/c5cc03557k 26051118

[46] Shen BQ, Xu K, Liu L, Raab H, Bhakta S, Kenrick M, Parsons-Reponte KL, Tien J, Yu SF, Mai E, Li D. Conjugation site modulates the in vivo stability and therapeutic activity of antibody-drug conjugates. Nature biotechnology. 2012 2 1;30(2):184–9. 10.1038/nbt.2108 22267010

[47] Wani MC, Taylor HL, Wall ME, Coggon P, McPhail AT. Plant antitumor agents. VI. Isolation and structure of taxol, a novel antileukemic and antitumor agent from Taxus brevifolia. Journal of the American Chemical Society. 1971 5;93(9):2325–7. 5553076 10.1021/ja00738a045

[48] Schiff PB, Fant J, Horwitz SB. Promotion of microtubule assembly in vitro by taxol. 10.1038/277665a0 423966

[49] Li C, Yu D, Inoue T, Yang DJ, Milas L, Hunter NR, Kim EE, Wallace S. Synthesis and evaluation of water-soluble polyethylene glycol-paclitaxel conjugate as a paclitaxel prodrug. Anti-cancer drugs. 1996 8 1;7(6):642–8. 8913432 10.1097/00001813-199608000-00004

[50] Bradley MO, Swindell CS, Anthony FH, Witman PA, Devanesan P, Webb NL, Baker SD, Wolff AC, Donehower RC. Tumor targeting by conjugation of DHA to paclitaxel. Journal of controlled release. 2001 7 6;74(1):233–6. 11489499 10.1016/s0168-3659(01)00321-2

[51] Luo Y, Prestwich GD. Synthesis and selective cytotoxicity of a hyaluronic acid-antitumor bioconjugate. Bioconjugate chemistry. 1999 9 20;10(5):755–63. 10502340 10.1021/bc9900338

[52] Chen X, Plasencia C, Hou Y, Neamati N. Synthesis and biological evaluation of dimeric RGD peptide-paclitaxel conjugate as a model for integrin-targeted drug delivery. Journal of medicinal chemistry. 2005 2 24;48(4):1098–106. 15715477 10.1021/jm049165z

[53] Ryppa C, Mann-Steinberg H, Biniossek ML, Satchi-Fainaro R, Kratz F. In vitro and in vivo evaluation of a paclitaxel conjugate with the divalent peptide E-[c (RGDfK) 2] that targets integrin α v β 3. International journal of pharmaceutics. 2009 2 23;368(1):89–97. 18992308 10.1016/j.ijpharm.2008.09.055

[54] Li C, Yu DF, Newman RA, Cabral F, Stephens LC, Hunter N, Milas L, Wallace S. Complete regression of well-established tumors using a novel water-soluble poly (L-glutamic acid)-paclitaxel conjugate. Cancer Research. 1998 6 1;58(11):2404–9. 9622081

[55] Gradishar WJ. Albumin-bound paclitaxel: a next-generation taxane. Expert opinion on pharmacotherapy. 2006 6 1;7(8):1041–53. 16722814 10.1517/14656566.7.8.1041

[56] Whelan Jo. "Targeted taxane therapy for cancer." Drug Discovery Today 7 2 [2002]: 90–92. 11790612 10.1016/s1359-6446(01)02149-3

[57] Guillemard V, Saragovi H. Taxane-antibody conjugates afford potent cytotoxicity, enhanced solubility, and tumor target selectivity. Cancer Research. 2001; 61(2);694–699. 11212270

[58] Safavy A, Bonner J, Waksal H, Buchsbaum D, Gillespie G, Arani R et al Synthesis and biological evaluation of paclitaxel−C225 conjugate as a model for targeted drug delivery. Bioconjugate Chem. 2003;14(2):302–310. 10.1021/bc020033z 12643740

[59] Quiles S, Raisch K, Sanford L, Bonner J, Safavy A. Synthesis and preliminary biological evaluation of high-drug-load paclitaxel-antibody conjugates for tumor-targeted chemotherapy. J Med Chem. 2010;53(2):586–594. 10.1021/jm900899g 19958000 PMC2841394

[60] Hammarström S. The carcinoembryonic antigen (CEA) family: structures, suggested functions and expression in normal and malignant tissues. Seminars in Cancer Biology. 1999;9(2):67–81. 10202129 10.1006/scbi.1998.0119

[61] Hefta S, Hefta L, Lee T, Paxton R, Shively J. Carcinoembryonic antigen is anchored to membranes by covalent attachment to a glycosylphosphatidylinositol moiety: identification of the ethanolamine linkage site. Proceedings of the National Academy of Sciences. 1988;85(13):4648–4652. 10.1073/pnas.85.13.4648 PMC280492 3387431

[62] Goldenberg M. Tumor localization and therapy with labeled anti-CEA antibody. U.S. Patent No. 4,348,376. 7 Sep. 1982.

[63] Rowland G, Simmonds R, Gore V, Marsden C, Smith W. Drug localisation and growth inhibition studies of vindesine-monoclonal anti-CEA conjugates in a human tumour xenograft. Cancer Immunol Immunother. 1986;21(3). 10.1007/BF00199359 PMC11038923 2421898

[64] Pedley R, Boden J, Boden R, Dale R, Begent R. Comparative radioimmunotherapy using intact or F(ab')2 fragments of 131I anti-CEA antibody in a colonic xenograft model. British Journal of Cancer. 1993;68(1):69–73. 8318423 10.1038/bjc.1993.288 PMC1968289

[65] Senba T, Kuroki M, Arakawa F, Yamamoto T, Kuwahara M, Haruno M et. al Tumor growth suppression by a mouse/human chimeric anti-CEA antibody and lymphokine-activated killer cells in vitro and in SCID mouse xenograft model." Anticancer Research 1998;18(1A):17–24 9568050

[66] Hoffer P, Bekerman C, Refetoff S, Fang V, Lathrop K. Radioiodinated antibody to carcinoembryonic Antigen (CEA). A potentially tumor specific scanning agent. Investigative Radiology. 1973;8(4):288.

[67] Primus FJ, MacDonald R, Goldenberg DM, Hansen HJ. Localization of GW-39 human tumors in hamsters by affinity-purified antibody to carcinoembryonic antigen." Cancer Research. 1977;37 5: 1544–1547. 851962

[68] Goldenberg D. Carcinoembryonic Antigen as a target cancer antigen for radiolabeled antibodies: prospects for cancer imaging and therapy. Tumor Biology. 1995;16(1):62–73. 7863224 10.1159/000217930

[69] Mach J, Carrel S, Forni M, Ritschard J, Donath A, Alberto P. Tumor localization of radio-labeled antibodies against carcinoembryonic antigen in patients with carcinoma. New England Journal of Medicine. 1980;303(1):5–10. 7189578 10.1056/NEJM198007033030102

[70] Buchegger F. Radiolabeled fragments of monoclonal antibodies against carcinoembryonic antigen for localization of human colon carcinoma grafted into nude mice. Journal of Experimental Medicine. 1983;158(2):413–427. 6886623 10.1084/jem.158.2.413 PMC2187333

[71] Sharkey R, Goldenberg D, Murthy S, Siegel J, Izon D, Gascon P et al Tumor targeting in patients with a second generation, high-affinity, anti-carcinoembryonic antigen (CEA) murine monoclonal antibody. Journal of Immunotherapy. 1992;11(2):144.

[72] Ito S, Muguruma N, Kusaka Y, Tadatsu M, Inayama K, Musashi Y et al Detection of human gastric cancer in resected specimens using a novel infrared fluorescent anti-human carcinoembryonic antigen antibody with an infrared fluorescence endoscope in vitro. Endoscopy. 2001;33(10):849–853. 11571680 10.1055/s-2001-17328

[73] Kaushal S, McElroy M, Luiken G, Talamini M, Moossa A, Hoffman R et al Fluorophore-conjugated anti-CEA Antibody for the intraoperative imaging of pancreatic and colorectal cancer. J Gastrointest Surg. 2008;12(11):1938–1950. 10.1007/s11605-008-0581-0 18665430 PMC4396596

[74] Metildi C, Kaushal S, Luiken G, Talamini M, Hoffman R, Bouvet M. Fluorescently labeled chimeric anti-CEA antibody improves detection and resection of human colon cancer in a patient-derived orthotopic xenograft (PDOX) nude mouse model. Journal of Surgical Oncology. 2013;109(5):451–458. 10.1002/jso.23507 24249594 PMC3962702

[75] Metildi C, Kaushal S, Pu M, Messer K, Luiken G, Moossa A et al Fluorescence-guided surgery with a fluorophore-conjugated antibody to carcinoembryonic antigen (CEA), that highlights the tumor, improves surgical resection and increases survival in orthotopic mouse models of human pancreatic cancer. Annals of Surgical Oncology. 2014;21(4):1405–1411. 10.1245/s10434-014-3495-y 24499827 PMC4334378

[76] Hiroshima Y, Maawy A, Metildi C, Zhang Y, Uehara F, Miwa S et al Successful fluorescence-guided surgery on human colon cancer patient-derived orthotopic xenograft mouse models using a fluorophore-conjugated anti-CEA antibody and a portable imaging system. Journal of Laparoendoscopic & Advanced Surgical Techniques. 2014;24(4):241–247. 24494971 10.1089/lap.2013.0418 PMC4047993

[77] Metildi C, Kaushal S, Luiken G, Hoffman R, Bouvet M. Advantages of fluorescence-guided laparoscopic surgery of pancreatic cancer labeled with fluorescent anti–carcinoembryonic antigen antibodies in an orthotopic mouse model. Journal of the American College of Surgeons. 2014;219(1):132–141. 10.1016/j.jamcollsurg.2014.02.021 24768506 PMC4065820

[78] Maawy A, Hiroshima Y, Kaushal S, Luiken G, Hoffman R, Bouvet M. Comparison of a chimeric anti-carcinoembryonic antigen antibody conjugated with visible or near-infrared fluorescent dyes for imaging pancreatic cancer in orthotopic nude mouse models. J Biomed Opt. 2013;18(12):126016 10.1117/1.JBO.18.12.126016 24356647 PMC3868446

[79] Cao HS, Kaushal S, Metildi CA, Menen RS, Lee C, Snyder CS, Messer K, Pu M, Luiken GA, Talamini MA, Hoffman RM. Tumor-Specific Fluorescent Antibody Imaging Enables Accurate Staging Laparoscopy in an Orthotopic Model of Pancreatic Cancer. Hepato-gastroenterology. 2012 9;59(118):1994 10.5754/hge11836 22369743 PMC4096574

[80] Maawy A, Hiroshima Y, Zhang Y, Luiken G, Hoffman R, Bouvet M. Polyethylene glycol (PEG) linked to near infrared (NIR) dyes conjugated to chimeric anti-carcinoembryonic antigen (CEA) antibody enhances imaging of liver metastases in a nude-mouse model of human colon cancer. PLoS ONE. 2014;9(5):e97965 10.1371/journal.pone.0097965 24859320 PMC4032229

[81] Maawy A, Hiroshima Y, Zhang Y, Luiken G, Hoffman R, Bouvet M. Specific tumor labeling enhanced by polyethylene glycol linkage of near infrared dyes conjugated to a chimeric anti-carcinoembryonic antigen antibody in a nude mouse model of human pancreatic cancer. J Biomed Opt. 2014;19(10):101504 10.1117/1.JBO.19.10.101504 24887695 PMC4160999

[82] Starling J, Hinson N, Marder P, Maciak R, Laguzza B, Corvalan J et al Development of a dual label fluorescence technique that can be utilized to elucidate the mechanism of action of monoclonal antibody-drug conjugates. Cancer Research. 1988;48(21):6211–6216. 3048656

[83] Safavy A, Georg G, Velde D, Raisch K, Safavy K, Carpenter M et al Site-specifically traced drug release and biodistribution of a paclitaxel−antibody conjugate toward improvement of the linker structure. Bioconjugate Chem. 2004;15(6):1264–1274. 10.1021/bc049868v 15546192

[84] Abe M, Kufe D. Effect of sodium butyrate on human breast carcinoma (MCF-7) cellular proliferation, morphology, and CEA production. Breast Cancer Res Tr. 1984;4(4):269–274. 10.1007/BF01806038 6518293

[85] Resnicoff M, Medrano E, Podhajcer O, Bravo A, Bover L, Mordoh J. Subpopulations of MCF-7 cells separated by Percoll gradient centrifugation: a model to analyze the heterogeneity of human breast cancer. Proceedings of the National Academy of Sciences. 1987;84(20):7295–7299. 10.1073/pnas.84.20.7295 PMC299279 2823256

[86] Correale P, Aquino A, Giuliani A, Pellegrini M, Micheli L, Cusi M et al Treatment of colon and breast carcinoma cells with 5-fluorouracil enhances expression of carcinoembryonic antigen and susceptibility to HLA-A(*)02.01 restricted, CEA-peptide-specific cytotoxic T cells in vitro. International Journal of Cancer. 2003;104(4):437–445. 12584740 10.1002/ijc.10969

[87] Casi G, Neri D. Antibody–drug conjugates: basic concepts, examples and future perspectives. Journal of controlled release. 2012 7 20;161(2):422–8. 10.1016/j.jconrel.2012.01.026 22306430

[88] Tsaltas G, Ford C, Gallant M. Demonstration of monoclonal anti-carcinoembryonic antigen (CEA) antibody internalization by electron microscopy, western blotting and radioimmunoassay. Anticancer Research. 1991;12 6B: 2133–2142. 1295459

[89] Shih LB, Goldenberg DM, Xuan H, Lu HW, Mattes MJ, Hall TC. Internalization of an intact doxorubicin immunoconjugate. Cancer Immunology, Immunotherapy. 1994 3 1;38(2):92–8. 8306371 10.1007/BF01526203 PMC11038197

[90] Ford CH, Tsaltas GC, Osborne PA, Addetia K. Novel Flow Cytometric Analysis of the Progress and Route of Internalization of Anti-Carcinoembryonic Antigen (CEA) Antibody. Cytometry. 1996;23:228–40. 8974868 10.1002/(SICI)1097-0320(19960301)23:3<228::AID-CYTO6>3.0.CO;2-E

[91] Schmidt M, Thurber G, Wittrup K. Kinetics of anti-carcinoembryonic antigen antibody internalization: effects of affinity, bivalency, and stability. Cancer Immunology, Immunotherapy. 2008;57(12):1879–1890. 10.1007/s00262-008-0518-1 18408925 PMC2840397

[92] Sato T, Sunamoto J, Ishii N, Koji T. Polysaccharide-coated immunoliposomes bearing anti-CEA Fab'fragment and their internalization by CEA-producing tumor cells. Journal of bioactive and compatible polymers. 1988 7 1;3(3):195–204.

[93] Hu CM, Kaushal S, Cao HS, Aryal S, Sartor M, Esener S, Bouvet M, Zhang L. Half-antibody functionalized lipid− polymer hybrid nanoparticles for targeted drug delivery to carcinoembryonic antigen presenting pancreatic cancer cells. Molecular pharmaceutics. 2010 4 26;7(3):914–20. 10.1021/mp900316a 20394436 PMC2884057

[94] He D, Yang H, Lin Q, Huang H. Arg 9-peptide facilitates the internalization of an anti-CEA immunotoxin and potentiates its specific cytotoxicity to target cells. The international journal of biochemistry & cell biology. 2005 1 31;37(1):192–205. 15381161 10.1016/j.biocel.2004.06.015

[95] Conway BR, Minor LK, Xu JZ, Gunnet JW, DeBiasio R, D'Andrea MR, Rubin R, DeBiasio R, Giuliano K, Zhou L, Demaresti KT. Quantification of G-protein coupled receptor internalization using G-protein coupled receptor-green fluorescent protein conjugates with the ArrayScan™ high-content screening system. Journal of biomolecular screening. 1999 4 1;4(2):75–86. 10838415 10.1177/108705719900400207

[96] Schlag BD, Lou Z, Fennell M, Dunlop J. Ligand dependency of 5-hydroxytryptamine 2C receptor internalization. Journal of Pharmacology and Experimental Therapeutics. 2004 9 1;310(3):865–70. 15113845 10.1124/jpet.104.067306

[97] Ghosh RN, Grove L, Lapets O. A quantitative cell-based high-content screening assay for the epidermal growth factor receptor-specific activation of mitogen-activated protein kinase. Assay and drug development technologies. 2004 10 1;2(5):473–81. 15671645 10.1089/adt.2004.2.473

[98] Ghosh RN, DeBiasio R, Hudson CC, Ramer ER, Cowan CL, Oakley RH. Quantitative cell-based high-content screening for vasopressin receptor agonists using Transfluor® technology. Journal of biomolecular screening. 2005 8 1;10(5):476–84. 16093557 10.1177/1087057105274896

[99] Fukunaga SI, Setoguchi S, Hirasawa A, Tsujimoto G. Monitoring ligand-mediated internalization of G protein-coupled receptor as a novel pharmacological approach. Life sciences. 2006 12 3;80(1):17–23. 16978657 10.1016/j.lfs.2006.08.022

[100] Wang J, Xie X. Development of a quantitative, cell‐based, high‐content screening assay for epidermal growth factor receptor modulators1. Acta Pharmacologica Sinica. 2007 10 1;28(10):1698–704. 17883960 10.1111/j.1745-7254.2007.00640.x

[101] Dragunow M. High-content analysis in neuroscience. Nature Reviews Neuroscience. 2008 10 1;9(10):779–88. 10.1038/nrn2492 18784656

[102] Sarkar S, Korolchuk VI, Renna M, Winslow AR, Rubinsztein DC. Methodological considerations for assessing autophagy modulators: a study with calcium phosphate precipitates. Autophagy. 2009 4 1;5(3):307–13. 19182529 10.4161/auto.5.3.7664

[103] Ross SL, Tran L, Winters A, Lee KJ, Plewa C, Foltz I, King C, Miranda LP, Allen J, Beckman H, Cooke KS. Molecular mechanism of hepcidin-mediated ferroportin internalization requires ferroportin lysines, not tyrosines or JAK-STAT. Cell metabolism. 2012 6 6;15(6):905–17. 10.1016/j.cmet.2012.03.017 22682226

[104] Lee SU, In HJ, Kwon MS, Park BO, Jo M, Kim MO, Cho S, Lee S, Lee HJ, Kwak YS, Kim S. β-Arrestin 2 Mediates G Protein-Coupled Receptor 43 Signals to Nuclear Factor-κB. Biological and Pharmaceutical Bulletin. 2013;36(11):1754–9. 23985900 10.1248/bpb.b13-00312

[105] Moreau K, Puri C, Rubinsztein DC. Methods to analyze SNARE-dependent vesicular fusion events that regulate autophagosome biogenesis. Methods. 2015 Mar 15;75:19–24. 10.1016/j.ymeth.2014.11.005 25461811 PMC4358838

[106] Grant BD, Donaldson JG. Pathways and mechanisms of endocytic recycling. Nature reviews Molecular cell biology. 2009 9 1;10(9):597–608. 10.1038/nrm2755 19696797 PMC3038567

[107] Matteoni R, Kreis TE. Translocation and clustering of endosomes and lysosomes depends on microtubules. The Journal of cell biology. 1987 9 1;105(3):1253–65. 3308906 10.1083/jcb.105.3.1253 PMC2114818

